# Toward better annotation in plant metabolomics: isolation and structure elucidation of 36 specialized metabolites from *Oryza sativa* (rice) by using MS/MS and NMR analyses

**DOI:** 10.1007/s11306-013-0619-5

**Published:** 2013-12-29

**Authors:** Zhigang Yang, Ryo Nakabayashi, Yozo Okazaki, Tetsuya Mori, Satoshi Takamatsu, Susumu Kitanaka, Jun Kikuchi, Kazuki Saito

**Affiliations:** 1 RIKEN Center for Sustainable Resource Science, 1-7-22 Suehiro, Tsurumi, Yokohama, Kanagawa 230-0045 Japan; 2 School of Pharmacy, Nihon University, 7-7-1 Narashinodai, Funabashi, Chiba 274-8555 Japan; 3 Graduate School of Pharmaceutical Sciences, Chiba University, 1-8-1 Inohana, Chiba, 260-8675 Japan; 4 Present Address: School of Pharmacy, Showa University, 1-5-8 Hatanodai, Shinagawa, Tokyo 142-8555 Japan

**Keywords:** *Oryza sativa*, Rice, Tandem mass spectrometry (MS/MS), Nuclear magnetic resonance (NMR), Specialized metabolites, Flavonoid

## Abstract

Metabolomics plays an important role in phytochemical genomics and crop breeding; however, metabolite annotation is a significant bottleneck in metabolomic studies. In particular, in liquid chromatography–mass spectrometry (MS)-based metabolomics, which has become a routine technology for the profiling of plant-specialized metabolites, a substantial number of metabolites detected as MS peaks are still not assigned properly to a single metabolite. *Oryza sativa* (rice) is one of the most important staple crops in the world. In the present study, we isolated and elucidated the structures of specialized metabolites from rice by using MS/MS and NMR. Thirty-six compounds, including five new flavonoids and eight rare flavonolignan isomers, were isolated from the rice leaves. The MS/MS spectral data of the isolated compounds, with a detailed interpretation of MS fragmentation data, will facilitate metabolite annotation of the related phytochemicals by enriching the public mass spectral data depositories, including the plant-specific MS/MS-based database, ReSpect.

**Electronic supplementary material:**

The online version of this article (doi:10.1007/s11306-013-0619-5) contains supplementary material, which is available to authorized users.

## Introduction

Metabolomics is an “omics” approach that allows researchers to chemically assign a set of metabolites in a given biological system (cell, tissue, or organism). In recent years, the use of metabolomics has rapidly developed in the fields of phytochemical genomics and crop breeding. It has been used in the investigation of plant biological mechanisms related to genetic and/or environmental factors (Roessner et al. 2001; Dixon et al. 2006; Saito and Matsuda 2010; Matsuda et al. 2012; Quanbeck et al. 2012; Saito 2013). Liquid chromatography (LC)–mass spectrometry (MS) is a common approach for metabolite identification using authentic standards by applying fragment patterns of tandem mass spectrometry (MS/MS) spectra in combination with the retention time or on-line UV spectrum data (Sumner et al. 2003; De Vos et al. 2007). High-resolution MS can provide an accurate mass of a molecule based on its precursor ion, and the specific fragment patterns of MS/MS can help reduce the number of potential molecular formulas for a metabolic peak, which can improve the speed and efficiency of metabolome studies (Matsuda et al. 2009; Sawada et al. 2012; Tautenhahn et al. 2012). Several MS/MS databases have been established to facilitate metabolite annotation, such as MassBank (http://www.massbank.jp/index.html?lang=en) (Horai et al. 2010), METLIN (http://metlin.scripps.edu/index.php) (Tautenhahn et al. 2012), HMDB (http://www.hmdb.ca/) (Wishart et al. 2009), LipidBlast (http://fiehnlab.ucdavis.edu/projects/LipidBlast/) (Kind et al. 2013), and ReSpect (http://spectra.psc.riken.jp) (Sawada et al. 2012). Moreover, databases for plant metabolomics have also been developed, which include KNApSAcK (http://kanaya.naist.jp/KNApSAcK/) (Afendi et al. 2012), MetaCyc (http://metacyc.org) (Zhang et al. 2005), PlantMetabolomics.org (http://www.plantmetabolomics.org) (Bais et al. 2010), KEGG (http://www.genome.jp/kegg/) (Kanehisa et al. 2010), and PRIMe (http://prime.psc.riken.jp/) (Sakurai et al. 2013).

Currently, metabolite identification is the major bottleneck in metabolomic studies (Matsuda et al. 2009; Dunn et al. 2012). It is estimated that there are over 200,000 to 1,000,000 different metabolites in the plant kingdom (Dixon and Strack 2003; Afendi et al. 2012). Many of plant secondary metabolites have been demonstrated to have ‘specialized’ roles for adaptive significance in protection against predator and microbial infection. Thus, these metabolites have recently been termed ‘specialized metabolites’, in addition, avoiding the impression of less important than ‘primary’ by the word ‘secondary’ (Pichersky and Lewinsohn 2011; Saito 2013). Identification of specialized metabolites still largely remains unknown, and many known metabolites are commercially unavailable. In untargeted metabolite profiling, most metabolites cannot be confidently identified due to the lack of authentic standards. These metabolites are often putatively annotated by comparison of their accurate mass and MS/MS patterns in the literature or databases (Sumner et al. 2007). However, the MS/MS spectra of plant-specialized metabolites in databases are especially limited. It is essential to acquire many MS/MS spectra to develop such databases. Isomers of many metabolites show similar chromatographic or mass spectrometric characteristics; therefore, substantial numbers of metabolites detected as MS peaks have not been unambiguously assigned to a single metabolite in MS-based metabolite profiling (Matsuda et al. 2009; Lei et al. 2011). Nuclear magnetic resonance (NMR) is a very powerful method for structural analysis, especially for stereoisomers. Hence, purification and structural elucidation of (un)known metabolites by combining a variety of spectroscopic methods such as MS/MS and NMR are useful for unambiguous identification of (un)known phytochemicals in plant metabolomics (Nakabayashi et al. 2009; Van der Hooft et al. 2013).

To enable better annotation in plant metabolomics, we aimed to isolate and identify specialized metabolites from model plants, like *Arabidopsis thaliana* (Nakabayashi et al. 2009), by using MS/MS and NMR methods. Recently, metabolome studies have attracted increasing attention in the case of *Oryza sativa* (rice) (Kusano et al. 2007; Suzuki et al. 2009; Calingacion et al. 2011; Redestig et al. 2011; Matsuda et al. 2012; Chen et al. 2013; Jung et al. 2013), which is one of the most important staple crops worldwide. Therefore, it is important to enrich the libraries of standard compounds and reference MS/MS spectra for specialized metabolites of rice. Habataki (indica variety) is one of elite Japanese cultivars, which has high yields. Previous studies have indicated that the rice leaves contain various flavonoids, and Habataki has high level production of a flavonoid *C*-glycoside (apigenin-6,8-di-*C*-α-l-arabinoside) due to the genetic polymorphism. Unequivocal structures of such metabolites are useful for understanding gene-to-metabolite correlations (Matsuda et al. 2012). In the present study, we performed isolation and identification of specialized metabolites from rice leaves (cultivar Habataki). On the basis of the accurate mass of the precursor ion and fragmentation patterns of collision-induced dissociation (CID) MS/MS, together with NMR spectra, 36 compounds, including five new flavonoids, were isolated and assigned from rice leaves. Most of the isolated compounds were flavonoid glycosides with tricin, apigenin, and chrysoeriol as the aglycones. The MS/MS data have been uploaded to the ReSpect database (http://spectra.psc.riken.jp), which will help to analyze metabolomic studies of rice and its related species, and facilitate the annotation of plant metabolites (Sawada et al. 2012).

## Materials and methods

### Plant material

Rice plants (cultivar Habataki) were grown in plastic pots containing granular soil (Bonsoru No.2; Sumitomo Chemical, Tokyo); after approximately 10 weeks of incubation, shoots were collected, lyophilized, and stored at −80 °C until use (Matsuda et al. 2012).

### Isolation of specialized metabolites

The leaf powder of rice (90 g) was extracted with 90 % methanol as described in a previous study (Matsuda et al. 2012). The extract was dissolved, suspended in water, and partitioned into a hexane and water layer. The water layer was subjected to ODS column chromatography and eluted with CH_3_OH–H_2_O (0:100 → 100:0 v/v; containing 0.05 % formic acid) to afford nine fractions (Fr.1–9). These fractions were purified using semipreparative HPLC performed under the following conditions: column, Cadenza CD-C18 or Unison UK-C18 columns, Imtakt 150 × 10 mm i.d.; particle size, 3 μm; solvents, water and methanol or acetonitrile, containing 0.1 % v/v formic acid; and flow rate, 3.0 mL/min. The following compounds were obtained: **1** (4.52 mg), **2** (12.69 mg), **3** (2.07 mg), **4** (2.57 mg), **5** (1.15 mg), **6** (0.94 mg), **7** (1.51 mg), **8** (1.23 mg), **9** (0.71 mg), **10** (2.53 mg), **11** (1.63 mg), **12** (3.93 mg), **13** (2.74 mg), **14** (0.58 mg), 1**5** (0.96 mg), **16** (1.89 mg), **17** (0.22 mg), **18** (0.09 mg), **19** (0.65 mg), **20** (0.20 mg), **21** (0.28 mg), **22** (0.64 mg), **23** (1.04 mg), **24** (0.76 mg), **25** (2.31 mg), **26** (2.20 mg), **27** (2.04 mg), **28** (1.25 mg), **29** (0.64 mg), **30** (0.29 mg), **31** (0.63 mg), **32** (2.29 mg), **33** (4.84 mg), **34** (4.99 mg), **35** (1.13 mg), and **36** (2.70 mg). For details regarding the isolation procedures from rice, see Supplementary data file S1.

### LC–quadrupole time-of-flight-tandem mass spectrometry (LC–QTOF-MS/MS) analysis

LC analysis was performed on the Waters ACQUITY UPLC™ System. Samples were injected into an ACQUITY bridged ethyl hybrid (BEH) C18 column (100 × 2.1 mm i.d., 1.7 μm; Waters, Milford, MA, USA), and the column temperature was set at 40 °C. The mobile phase consisted of A (0.1 % v/v formic acid in water) and B (0.1 % v/v formic acid in acetonitrile). The gradient conditions of the mobile phase were as follows: 0 min, 99.5 % A; 10.0 min, 20 % A; 10.01 min, 0.5 % A; 12.0 min, 0.5 % A; 12.1 min, 99.5 % A; and 14.5 min, 99.5 % A. The flow rate was 0.30 mL/min. UV–visible absorption spectra of samples were determined using a photodiode array (PDA) detector in the range of 200–600 nm. The sample injection volume was 1 μL.

MS detection was performed on a Waters Xevo G2 QTOF mass spectrometer with an electrospray ionization (ESI) interface (Waters). Full scan mass spectra were recorded through a range of 50–1,500 *m*/*z*. Nitrogen was used as the nebulizer and auxiliary gas; argon was utilized as the collision gas. The ESI source was operated in positive and negative ionization modes with a capillary voltage of 3 kV, sampling cone voltage of 25 V, cone gas flow of 50 L/h, desolvation gas flow of 800 L/h, desolvation temperature of 450 °C, source temperature of 120 °C, and CID energy ramped from 10 to 50 eV. Tandem MS analysis was performed using fast data directed analysis (FastDDA), which is rapid automated, intelligent MS/MS data acquisition for targeted qualitative analyses. Data acquisition and processing were performed with the MassLynx 4.1 software.

### NMR analysis

The NMR spectra were recorded on a Bruker 600 MHz spectrometer with a DCH CryoProbe (Bruker BioSpin GmbH, Rheinstetten, Germany). One-dimensional (1D) ^1^H-NMR was measured of 64 or 128 scans and at a receiver gain of 11.3 using standard pulse sequences. 1D ^13^C-NMR, and two-dimensional (2D) NMR experiments, ^1^H–^1^H correlation spectroscopy (COSY), ^1^H–^13^C heteronuclear single quantum coherence spectroscopy (HSQC), and ^1^H–^13^C heteronuclear multiple bond connectivity spectroscopy (HMBC) were obtained using standard pulse sequences. Dimethylsulfoxide-*d*_6_ or methanol-*d*_4_ was used as solvent, and tetramethylsilane (TMS) was used as an internal standard. The samples were added to 5 mm Shigemi micro NMR tubes (Shigemi, DMS-005B and MMS-005B, Tokyo). NMR data were acquired and processed with the TopSpin software (Bruker BioSpin GmbH, Rheinstetten, Germany).

### Data upload

All data acquired by LC–QTOF-MS/MS were uploaded to DROP Met in PRIMe (http://prime.psc.riken.jp/) and are freely available.

## Results and discussion

**Fig. 1:**
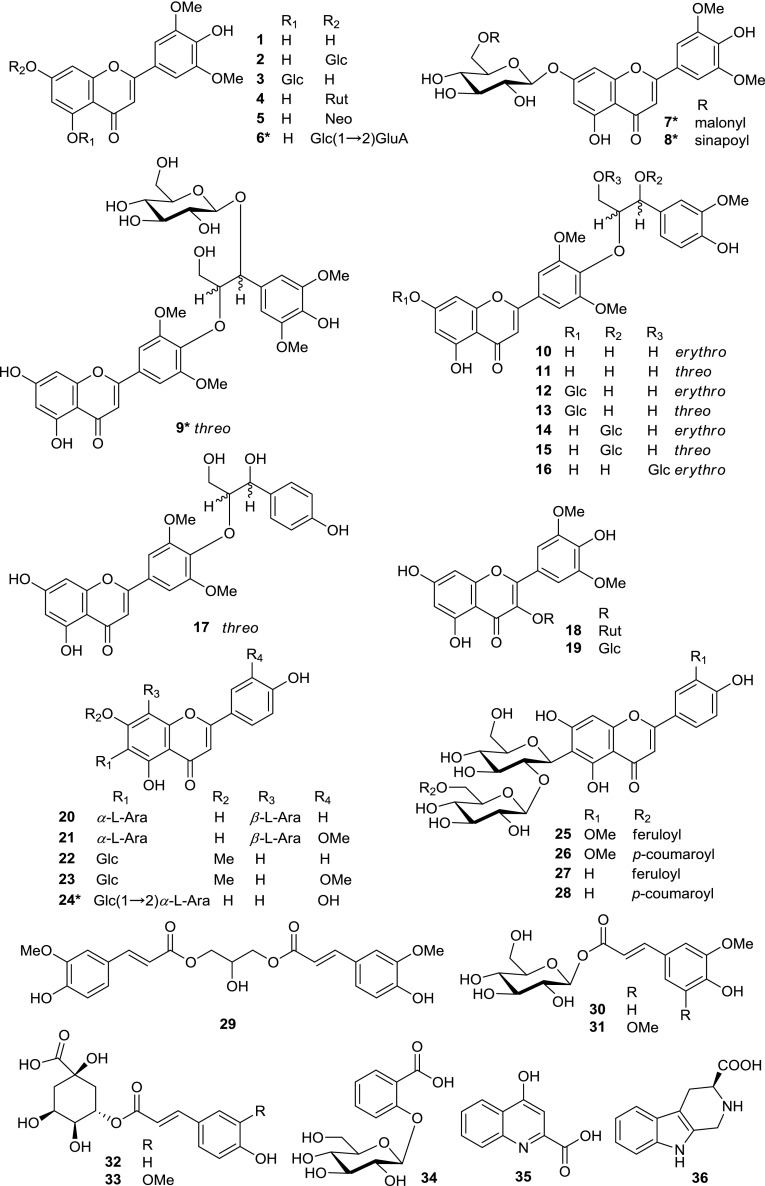
Structures of compounds **1**–**36**. *Glc* β-d-glucopyranosyl, *Rut* rutinosyl, *Neo* neohesperidosyl, *GluA* glucuronopyranosyl, *Ara* arabinosyl, *erythro* and *threo* the forms of lignan parts of flavonolignans, *asterisk* new compound

In this study, to achieve better metabolite identification, namely improving the metabolite annotation level in general metabolomics research community, we mainly focused on and selected the flavonoids and flavonolignans for further isolation and structure elucidation from initial LC–MS experiments, indicating those as the representative detectable metabolites. 36 compounds, including five new flavonoids (**6**–**9** and **24**), were isolated and assigned from the leaves of rice using MS/MS and NMR methods (Fig. 1). To our knowledge, this is the first time that 18 of the known compounds (**4**, **5**, **12**, **13**, **17**–**23**, **29**–**33**, **35**, and **36**) have been isolated from rice leaves. Those 36 compounds have been assigned in LC-PDA chromatogram of rice leaves extract (Supplementary Figure S1). Herein, we report the structural elucidation of new flavonoids and analysis of the MS/MS fragmentation patterns of isolated compounds by using high-resolution QTOF mass spectrometry with an ESI source. In the MS/MS analysis, the ramped collision energies mode was used to obtain a combined spectrum from fragments detected at various collision energies (Matsuda et al. 2009) because the fragmentation patterns observed in MS/MS spectra depend on many factors, including the mass spectrometer instrument and its operating conditions, especially collision energy. In addition, the structures of known compounds were identified by ^1^H, ^13^C-NMR analyses.

### Structure elucidation of new compounds **6**–**9** and **24**

**Fig. 2:**
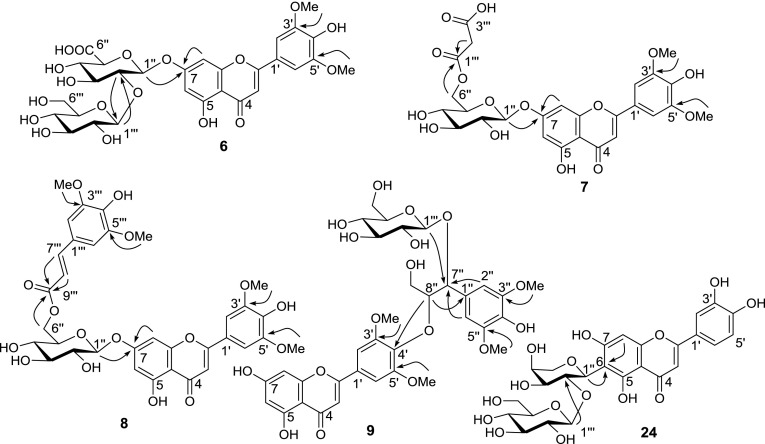
Key HMBC correlations of compounds **6**–**9** and **24**

Compound **6** was obtained as a yellow amorphous powder. The molecular formula of compound **6** was established as C_29_H_32_O_18_ by HR-ESI-QTOF-MS. The MS/MS spectra of the precursor ion at *m*/*z* 669 [M + H]^+^ gave a product ion at *m*/*z* 507 [(M + H)-162]^+^, corresponding to the loss of a hexose group, and a major product ion at *m*/*z* 331 [(M + H)-162-176]^+^, representing the loss of hexose and glucuronosyl groups. The ^1^H-NMR spectrum of compound **6** indicated an A_2_-type aromatic proton signal at *δ* 7.37 (2H, s); *meta*-coupled proton signals at *δ* 6.52 (1H, d, *J* = 2.0 Hz) and 7.01 (1H, d, *J* = 2.0 Hz); an aromatic proton signal at *δ* 7.07 (1H, s); two methoxy proton signals at *δ* 3.89 (6H, s); and two anomeric proton signals at *δ* 5.33 (1H, d, *J* = 5.7 Hz) and 4.48 (1H, d, *J* = 7.9 Hz) (Table 1). Furthermore, in combination with the ^13^C-NMR and 2D NMR (COSY, HSQC, and HMBC) spectra, these data indicated that compound **6** was tricin glucopyranosyl-glucuronopyranoside. The relatively large coupling constant values of anomeric protons suggested that the configuration of glucose and glucuronic acid were β forms. In addition, in the HMBC spectrum, the anomeric proton signals *δ* 5.33 (H-1′′) and 4.48 (H-1′′′) showed long-range correlation with the carbon signals at *δ* 162.5 (C-7) and 82.5 (C-2′′), respectively, suggesting that the glucuronosyl was located at the C-7 of aglycone and that glucose was located at the C-2 of glucuronosyl (Fig. 2). Based on these findings, compound **6** was assigned as tricin 7-*O*-(2′′-*O*-β-d-glucopyranosyl)-β-d-glucuronopyranoside.

**Table 1:** ^1^H- and ^13^C-NMR spectral data of compounds **6**, **7** and **8** [(600/150 MHz, in DMSO-*d*_6_, 25 °C, TMS, *δ* (ppm) (*J* = Hz)]

Position	**6**		**7**		**8**	
	*δ*_H_	*δ*_C_	*δ*_H_	*δ*_C_	*δ*_H_	*δ*_C_
2	–	164.1	–	164.0	–	163.9
3	7.07 (s)	103.7	7.06 (s)	103.9	6.96 (s)	103.3
4	–	182.0	–	181.8	–	181.9
4a	–	105.4	–	105.5	–	105.3
5	–	161.0	–	161.2	–	161.1
6	6.52 (d 2.0)	99.4	6.49 (br s)	99.4	6.52 (d 1.9)	99.3
7	–	162.5	–	162.7	–	162.6
8	7.01 (d 2.0)	95.7	6.86 (br s)	95.2	6.85 (d 1.9)	95.1
8a	–	156.7	–	156.9	–	156.6
1′	–	120.1	–	120.2	–	119.8
2′	7.37 (s)	104.7	7.37 (s)	104.6	7.28 (s)	104.3
3′	–	148.1	–	148.2	–	148.0
4′	–	140.0	–	140.0	–	140.1
5′	–	148.1	–	148.2	–	148.0
6′	7.37 (s)	104.7	7.37 (s)	104.6	7.28 (s)	104.3
3′,5′-OMe	3.89 (s)	56.3	3.89 (s)	56.4	3.88 (s)	56.2
5-OH	12.97 (br s)	–	13.05 (br s)	–	13.01 (br s)	–
1′′	5.33 (d 5.7)	98.3	5.10 (d 7.4)	99.7	5.15 (d 7.3)	99.5
2′′	3.55 (m)	82.5	3.30 (m)	73.0	3.32 (m)	72.9
3′′	3.19 (m)	77.0	3.32 (m)	76.1	3.37 (m)	76.2
4′′	3.44 (m)	70.9	3.17 (m)	69.6	3.26 (m)	70.0
5′′	3.98 (m)	74.6	3.75 (m)	73.8	3.82 (m)	73.7
6′′	–	170.0	4.34 (d 11.6)	63.8	4.57 (d 11.9)	63.2
			4.15 (dd 11.9, 6.5)		4.10 (dd 11.9, 7.3)	
1′′′	4.48 (d 7.9)	104.7	–	167.4	–	124.1
2′′′	2.99 (m)	74.6	3.27 (s)	42.3	6.80 (s)	105.7
3′′′	3.16 (m)	76.1	–	167.7	–	147.8
4′′′	3.10 (m)	69.6	–	–	–	138.1
5′′′	3.55 (m)	75.0	–	–	–	147.8
6′′′	3.53 (m)	60.6	–	–	6.80 (s)	105.7
	3.44 (m)					
7′′′	–	–	–	–	7.47 (d 15.9)	145.4
8′′′	–	–	–	–	6.44 (d 15.9)	114.3
9′′′	–	–	–	–	–	166.2
3′′′,5′′′-OMe	–	–	–	–	3.71 (s)	55.7

*s* Singlet, *m* multilet, *d* doublet, *dd* double doublet, *br s* broad singlet

Compound **7** was obtained as a yellow amorphous powder. HR-ESI-QTOF-MS gave the molecular formula C_26_H_26_O_15_. The MS/MS spectra of the precursor ion at *m*/*z* 579 [M + H]^+^ showed a product ion at *m*/*z* 493 [(M + H)-86]^+^, corresponding to the loss of a malonyl group, and a major product ion at *m*/*z* 331 [(M + H)-86-162]^+^, corresponding to the loss of malonyl and hexose groups. The ^1^H-NMR spectrum of compound **7** indicated an A_2_-type aromatic proton signal at *δ* 7.37 (2H, s); three aromatic proton signals at *δ* 6.45 (1H, brs), 6.73 (1H, brs), and 7.06 (1H, s); two methoxy proton signals at *δ* 3.89 (6H, s); and a sugar of the anomeric proton signal at *δ* 5.10 (1H, d, *J* = 7.4 Hz) (Table 1). These data, together with the ^13^C-NMR and 2D NMR (COSY, HSQC, and HMBC) spectra, indicated that compound **7** was tricin malonyl-glucopyranoside. Furthermore, in the HMBC spectrum, the anomeric proton signal *δ* 5.10 (H-1′′) showed long-range correlation with the carbon signal at *δ* 162.7 (C-7), suggesting that the glucose was located at C-7. The sugar proton signal at *δ* 4.15 (H-6′′) showed correlation with the carbon signal at *δ* 167.4 (C-1′′′), suggesting that the malonyl moiety was located at the C-6 of glucose (Fig. 2). Thus, compound **7** was assigned as tricin 7-*O*-(6′′-*O*-malonyl)-β-d-glucopyranoside.

Compound **8** was obtained as a yellow amorphous powder. The molecular formula of compound **8** was determined as C_34_H_34_O_16_ with HR-ESI-QTOF-MS. The MS/MS spectra of the precursor ion at *m*/*z* 699 [M + H]^+^ gave a major product ion at *m*/*z* 331 [(M + H)-206-162]^+^, representing the loss of sinapoyl and hexose groups. The fragment ion of the sinapoyl moiety at *m/z* 207 was also observed (Cuyckens and Claeys 2004). The ^1^H-NMR spectrum of compound **8** indicated an A_2_-type aromatic proton signal at *δ* 7.28 (2H, s); *meta*-coupled proton signals at *δ* 6.52 (1H, d, *J* = 1.9 Hz) and 6.89 (1H, d, *J* = 1.9 Hz); an aromatic proton signal at *δ* 6.96 (1H, s); two methoxy proton signals at *δ* 3.88 (6H, s); and an anomeric proton signal at *δ* 5.15 (1H, d, *J* = 7.3 Hz), which were similar to those of compounds **6** and **7** (Table 1). In addition, we observed an A_2_-type aromatic proton signal at *δ* 6.80 (2H, s); two methoxy proton signals at *δ* 3.71 (6H, s); and two olefinic proton signals at *δ* 6.44 (1H, d, *J* = 15.9 Hz) and 7.47 (1H, d, *J* = 15.9 Hz), suggesting the presence of a *trans*-sinapoyl moiety. Furthermore, in the HMBC spectrum, the anomeric proton signal *δ* 5.15 (H-1′′) showed a long-range correlation with the carbon signal at *δ* 162.6 (C-7), suggesting that the glucose was located at C-7. The sugar proton signal at *δ* 4.10 (H-6′′) showed correlation with the carbon signal at *δ* 166.2 (C-9′′′), suggesting that the sinapoyl moiety was located at the C-6 of glucose (Fig. 2). Thus, compound **8** was assigned as tricin 7-*O*-(6′′-(*E*)-sinapoyl)-β-d-glucopyranoside.

Compound **9** was obtained as a yellow amorphous powder. The molecular formula of compound **9** was established as C_34_H_38_O_17_ by HR-ESI-QTOF-MS. The MS/MS spectra of the precursor ion at *m*/*z* 719 [M + H]^+^ gave a product ion at m/z 557 [(M + H)-162]^+^, corresponding to the loss of a hexose group, and a product ion at *m*/*z* 331 [(M + H)-226-162]^+^, corresponding to the loss of syringylglyceryl and hexose groups. The product ion at *m/z* 539 was formed by the loss of glucose and a water molecule from the precursor ion at *m/z* 719. In addition, a major product ion was observed at *m*/*z* 209, which was formed by the loss of a water molecule from the syringylglyceryl moiety. The ^1^H-NMR spectrum of compound **9** indicated an A_2_-type aromatic proton signal at *δ* 7.26 (2H, s); *meta*-coupled proton signals at *δ* 6.10 (1H, d, *J* = 2.0 Hz) and 6.34 (1H, d, *J* = 2.0 Hz); an aromatic proton signal at *δ* 6.64 (1H, s); two methoxy proton signals at *δ* 3.96 (6H, s); and an anomeric proton signal at *δ* 4.57 (1H, d, *J* = 7.5 Hz). Moreover, the ^1^H-NMR spectrum of compound **9** was similar to that of compound **15**, except for an A_2_-type aromatic proton signal at *δ* 6.81 (2H, s) and six proton signals at *δ* 3.84 (6H, s), corresponding to two methoxyl groups of the syringylglyceryl moiety (Table 2). Furthermore, in combination with the ^13^C-NMR and 2D NMR (COSY, HSQC, and HMBC) spectra, these data indicated that compound **9** was a flavonolignan glycoside with tricin as the aglycone. In addition, the coupling constant of *J*_H-7′′, H-8′′_ was 5.5 Hz, suggesting that compound **9** was of the *threo* type because the coupling constant between the adjacent protons of the *threo* form is known to be larger than that of the *erythro* form (Bouaziz et al. 2002). To determine the absolute configuration of the syringylglyceryl and guaiacylglyceryl moieties of flavonolignans **9**–**17**, we measured the circular dichroism (CD) spectra. However, these compounds did not exhibit Cotton effects, presumably due to conformational mobility (Wenzig et al. 2005). Furthermore, in the HMBC spectrum, the anomeric proton signal *δ* 4.57 (H-1′′′) showed long-range correlation with the carbon signal at *δ* 82.0 (C-7′′), suggesting that the glucose was located at C-7′′. The syringylglyceryl proton signal at *δ* 4.55 (H-8′′) showed correlation with the carbon signal at *δ* 140.7 (C-4′), suggesting that the location of the syringylglyceryl moiety was at C-4′ (Fig. 2). Thus, compound **9** was assigned as tricin 4′-*O*-(*threo*-β-syringylglyceryl) ether 7′′-*O*-β-d-glucopyranoside.

**Table 2:** ^1^H- and ^13^C-NMR spectral data of compounds **9** and **24** [(600/150 MHz, TMS, *δ* (ppm) (*J* = Hz)]

Position	**9** ^a^		**24** ^b^	
	*δ*_H_	*δ*_C_	*δ*_H_	*δ*_C_
2	–	165.3	–	163.5
3	6.64 (s)	106.08	6.56 (s)	102.6
4	–	183.9	–	181.6
4a	–	105.5	–	103.1
5	–	163.4	–	156.5
6	6.10 (d 2.0)	100.4	–	108.3
7	–	166.4	–	163.9
8	6.34 (d 2.0)	95.3	6.37 (s)	93.6
8a	–	159.6	–	160.7
1′	–	128.1	–	121.4
2′	7.26 (s)	105.3	7.375 (s)	113.1
3′	–	154.8	–	145.8
4′	–	140.7	–	150.0
5′	–	154.8	6.87 (d 8.0)	116.0
6′	7.26 (s)	105.3	7.382 (d 8.0)	118.8
3′,5′-OMe	3.96 (s)	57.1	–	
5-OH	–	–	13.55 (br s)	
1′′	–	130.9	4.58 (d 9.4)	72.2
2′′	6.81 (s)	106.13	4.60 (m)	78.7
3′′	–	148.9	3.61 (d 5.9)	74.5
4′′	–	136.2	3.80 (m)	68.6
5′′	–	148.9	3.75 (d 11.5)	70.2
			3.52 (d 11.7)	
6′′	6.81 (s)	106.13	–	–
7′′	5.17 (d 5.5)	82.0	–	–
8′′	4.55 (m)	86.9	–	–
9′′	3.72 (dd 12.1, 4.3)	61.8	–	–
	3.39 (m)			
3′′,5′′-OMe	3.84 (s)	56.9	–	–
1′′′	4.57 (d 7.5)	104.9	4.19 (d 7.7)	104.9
2′′′	4.33 (m)	75.7	2.87 (t 8.3, 8.6)	74.6
3′′′	3.42 (m)	78.2	3.06 (t 8.9)	76.5
4′′′	3.19 (m)	71.5	2.97 (t 8.9, 9.3)	69.8
5′′′	3.37 (m)	77.9	2.70 (t 9.3)	76.2
6′′′	3.75 (dd 11.9, 2.3)	62.6	3.16 (m)	60.8
	3.60 (dd 11.9, 5.3)			

*s* Singlet, *m* multilet, *d* doublet, *dd* double doublet, *br s* broad singlet, *t* triplet

^a^in CD_3_OD, 25 °C

^b^in DMSO-*d*_6_, 45 °C

Compound **24** was obtained as a yellow amorphous powder. The molecular formula was found to be C_26_H_28_O_15_ by HR-ESI-QTOF-MS. The MS/MS spectra of the precursor ion at *m*/*z* 581 [M + H]^+^ showed a product ion at *m*/*z* 419 [(M + H)-162]^+^, corresponding to the loss of a hexose group. In addition, characteristic fragment ions of *C*-glycoside were also observed. The fragment ions at *m/z* 401, 383, and 365 were formed by the loss of water molecules from the *C*-glycoside fragment at *m/z* 419. Product ions at *m/z* 329 [(M + H)-162-90]^+^ (^0.2^X^+^) and 353 [(M + H)-162-66]^+^ (^0.4^X^+^-2H_2_O) were formed by cross-ring cleavages of a sugar residue from *m/z* 419. The ^1^H-NMR spectrum of compound **24** indicated *ortho*-coupled proton signals at *δ* 6.87 (1H, d, *J* = 8.0 Hz) and 7.382 (1H, d, *J* = 8.0 Hz); three aromatic proton signals at *δ* 6.37 (1H, s), 6.56 (1H, s), and 7.375 (1H, s); and two anomeric proton signals at *δ* 4.58 (1H, d, *J* = 9.4 Hz) and 4.19 (1H, d, *J* = 7.7 Hz) (Table 2). Furthermore, in combination with the ^13^C-NMR and 2D NMR (COSY, HSQC, and HMBC) spectra, these data indicated that compound **24** was luteolin glucopyranosyl-arabinoside. The relatively large coupling constant values of anomeric protons suggested that the configuration of the glucose was the *β* form and of the arabinose was the *α* form (Xie et al. 2003). In addition, in the HMBC spectrum, the anomeric proton signals *δ* 4.58 (H-1′′) and 4.19 (H-1′′′) showed a long-range correlation with the carbon signals at *δ* 108.3 (C-6) and 78.7 (C-2′′), respectively, suggesting that the arabinosyl moiety was located at the C-6 of aglycone and glucosyl at the C-2 of arabinose (Fig. 2). Based on these data, compound **24** was assigned as luteolin 6-*C*-(2′′-*O*-β-d-glucopyranosyl)-α-l-arabinoside.

### Structure identification of known compounds **1**–**5**, **10**–**23**, and **25**–**36**

#### Flavonoids

MS spectra of compound **1** in the positive and negative ionization modes showed a protonated molecular ion at *m/z* 331 and a deprotonated molecular ion at *m/z* 329, respectively. MS/MS spectra of compounds **2**–**5** and **10**–**17** in the positive mode showed diagnostic fragment ions of tricin at *m*/*z* 331, suggesting that these compounds were tricin derivatives. For the detailed MS/MS analysis of these compounds, see Sect. 3.3. Furthermore, on comparison of the ^1^H- and ^13^C-NMR spectral data (Supplementary data file S2) with those in the literature, these compounds were assigned as tricin (**1**) (Jiao et al. 2007), tricin 7-*O*-β-d-glucopyranoside (**2**) (Kong et al. 2007), tricin 5-*O*-β-d-glucopyranoside (**3**) (Adjei-Afriyie et al. 2000), tricin 7-*O*-rutinoside (**4**) (Hirai et al. 1986), tricin 7-*O*-neohesperidoside (**5**) (Zhang et al. 2009), tricin 4′-*O*-(*erythro*-β-guaiacylglyceryl) ether (**10**) (Bouaziz et al. 2002), tricin 4′-*O*-(*threo*-β-guaiacylglyceryl) ether (**11**) (Bouaziz et al. 2002), tricin 4′-*O*-(*erythro*-β-guaiacylglyceryl) ether 7-*O*-β-d-glucopyranoside (**12**) (Bouaziz et al. 2002), tricin 4′-*O*-(*threo*-β-guaiacylglyceryl) ether 7-*O*-β-d-glucopyranoside (**13**) (Bouaziz et al. 2002), tricin 4′-*O*-(*erythro*-β-guaiacylglyceryl) ether 7′′-*O*-β-d-glucopyranoside (**14**) (Baek et al. 2012), tricin 4′-*O*-(*threo*-β-guaiacylglyceryl) ether 7′′-*O*-β-d-glucopyranoside (**15**) (Baek et al. 2012), tricin 4′-*O*-(*erythro*-β-guaiacylglyceryl) ether 9′′-*O*-β-d-glucopyranoside (**16**) (Baek et al. 2012), and tricin 4′-*O*-(*threo*-β-4-hydroxyphenylglyceryl) ether (**17**) (Chang et al. 2010).

The MS spectra of compounds **18** and **19** in the positive ionization mode showed protonated molecular ions at *m/z* 509 and 655, respectively. The MS/MS spectra of compound **18** showed a major fragment ion at *m*/*z* 347 [(M + H)-162]^+^, corresponding to the loss of a hexose group. MS/MS spectra of compound **19** showed major fragment ions at *m*/*z* 509 [(M + H)-146]^+^ and 347 [(M + H)-146-162]^+^, indicating the loss of deoxyhexose and hexose groups. Furthermore, comparing the ^1^H-NMR spectral data with those in the literature, they were assigned as syringetin 3-*O*-β-d-glucopyranoside (**18**) (Guo et al. 2010) and syringetin 3-*O*-rutinoside (**19**) (Victoire et al. 1988).

The MS/MS spectra of compounds **20**–**23** and **25**–**28** showed characteristic fragment ions of *C*-glycosides. For the detailed MS/MS analysis of these compounds, see Sect. 3.3. Comparing the ^1^H-NMR spectral data with those in the literature, four flavonoid *C*-glycosides (compounds **20**–**23**) were assigned as apigenin 6-*C*-α-l-arabinosyl-8-*C*-β-l-arabinoside (**20**) (Xie et al. 2003), chrysoeriol 6-*C*-α-l-arabinosyl-8-*C*-β-l-arabinoside (**21**) (Shie et al. 2010), swertisin (**22**) (Cheng et al. 2000), and isoorientin 7,3′-dimethyl ether (**23**) (Zhu et al. 2010). Moreover, comparing the ^1^H- and ^13^C-NMR spectral data with those in the literature, four *O,C-*glycosides (compounds **25**–**28**) were assigned as isoscoparin 2′′-*O*-(6′′′-(*E*)-feruloyl)-glucopyranoside (**25**) (Besson et al. 1985), isoscoparin 2′′-*O*-(6′′′-(*E*)-*p*-coumaroyl)-glucopyranoside (**26**) (Besson et al. 1985), isovitexin 2′′-*O*-(6′′′-(*E*)-feruloyl)-glucopyranoside (**27**) (Markham et al. 1998), and isovitexin 2′′-*O*-(6′′′-(*E*)-*p*-coumaroyl)-glucopyranoside (**28**) (Markham et al. 1998).

#### Phenylpropanoids and salicylic acid glycoside

The MS spectra of compounds **29**, **30**, **31**, **32**, and **33** in the negative ionization mode showed precursor ions at *m/z* 443, 355, 385, 337, and 367, respectively. The MS/MS spectra of compounds **29**, **30**, and **33** gave the same characteristic fragment ions at *m*/*z* 193 [ferulic acid-H]^-^, indicating the presence of a feruloyl moiety in these compounds. Similarly, in the MS/MS spectra of compounds **31** and **32**, fragment ions of sinapic acid were observed at *m/z* 223 and of coumaric acid at *m/z* 163. On comparing the ^1^H- and ^13^C-NMR spectral data with those in the literature, these compounds were assigned as 1,3-*O*-diferuloylglycerol (**29**) (Luo et al. 2012), 1-*O*-feruloyl-β-d-glucose (**30**) (Miyake et al. 2007), 1-*O*-sinapoyl-β-d-glucose (**31**) (Miyake et al. 2007), 3-*O*-*p*-coumaroylquinic acid (**32**) (Ma et al. 2007), and 3-*O*-feruloylquinic acid (**33**) (Ida et al. 1994). The MS spectra of compound **34** in the negative ionization mode showed a deprotonated molecular ion at *m/z* 299. The MS/MS spectra of the precursor ion at *m/z* 299 gave a major fragment ion at *m*/*z* 137 [(M − H)-162]^−^, suggesting the presence of a hexose group. Compound **34** was assigned as salicylic acid 2-*O*-β-d-glucopyranoside (Grynkiewicz et al. 1993) by comparing the ^1^H- and ^13^C-NMR spectral data with those in the literature.

#### Alkaloids

The MS spectra of compound **35** in the positive and negative ionization modes showed precursor ions at *m/z* 190 and 188, respectively. The MS/MS spectra of the precursor ion at *m/z* 190 produced major fragment ions at *m*/*z* 172 and 144. This compound was assigned as kynurenic acid (**35)** (Beretta et al. 2007) by comparing the MS/MS and ^1^H-, ^13^C-NMR spectral data with those in the literature. The MS spectra of compound **36** in the positive and negative ionization modes showed a protonated molecular ion at *m/z* 217 and a deprotonated molecular ion at *m/z* 215, respectively. On comparing the ^1^H-NMR spectral data with those in the literature, compound **36** was assigned as lycoperodine-1 (Yahara et al. 2004).

### MS/MS data acquisition of isolated compounds

Certain classes of specialized metabolites with similar structures in plants show characteristic fragments or neutral losses in their MS/MS spectra. Flavonoids, a major class of plant-specialized metabolites, include subclasses such as flavonol, flavone, flavan-3-ol, isoflavone, and anthocyanin. Many flavonoids are positional isomers or homologues, which have a basic C6-C3-C6 skeleton, with two aromatic rings linked by a three-carbon chain (Dixon and Steele 1999). Flavonoids are commonly present as *O*- or *C*-glycosides. The flavonoid *O*-glycosides usually have sugar moieties bonded to the 4′-, 3-, and 7-hydroxyl groups of the aglycone. The flavonoid *C*-glycosides have sugar substituents directly linked to the aglycone by C–C bonds. The C-6 and C-8 positions are the common locations in *C*-glycosides. The flavonoid *O,C-*glycosides have sugar moieties linked to the hydroxyl group of the aglycone or *C*-glycosyl residue. Numerous flavonoid glycosides have been identified or characterized using the LC–MS approach (Cuyckens and Claeys 2004; de Rijke et al. 2006; Farag et al. 2007; Kachlicki et al. 2008; Van der Hooft et al. 2012; Wojakowska et al. 2013). To aid in the annotation of phytochemicals, we have reported the characteristic MS/MS fragmentation patterns of the isolated compounds.

#### Fragmentation of flavonoid O-glycosides and flavonolignans

In the MS/MS analysis of *O*-glycosides, the neutral loss of hexose (*m/z* 162) and deoxyhexose (*m/z* 146) from the precursor ion are common fragmentations, which are formed by rearrangement reactions at the interglycosidic bonds (Cuyckens and Claeys 2004). Here, we focused on the comparison of fragmentation patterns of isobaric and isomeric flavonoid *O*-glycosides and flavonolignans.

Tricin 5-*O*-β-D-glucopyranoside (**3**) showed a higher relative abundance of a tricin aglycone fragment ion at *m/z* 331 [(M + H)-162]^+^ (Y_0_^+^) than tricin 7-*O*-β-d-glucopyranoside (**2**) in their MS spectra and lower relative abundance of ions at *m/z* 493 than compound **2** in the MS and MS/MS spectra (Supplementary Figure S2). These results suggested that the glucose at the 5-position was lost more readily than at the 7-position. Our results are in agreement with earlier studies on luteolin 5-*O*-glucoside and luteolin 7-*O*-glucoside (Grayer et al. 2000).

The MS/MS spectra of tricin 7-*O*-rutinoside (**4**) in the positive ionization mode showed a major fragment ion at *m/z* 493 [(M + H)-146]^+^ (Y_1_^+^), which was formed by the loss of rhamnose, whereas tricin 7-*O*-neohesperidoside (**5**) produced only a very low abundance of the Y_1_^+^ ion. Compounds **4** and **5** both showed the base peak of aglycone fragment ions at *m/z* 331 (Y_0_^+^), which were formed by the loss of rutinose and neohesperidose moieties, respectively. These results indicated that the Y_0_^+^/Y_1_^+^ ratio was higher for 1 → 2 linked neohesperidose [rhamnosyl (1 → 2)-glucose] than for 1 → 6 linked rutinose [rhamnosyl (1 → 6)-glucose] in the positive ionization mode (Ma et al. 2001). However, in the negative ionization mode, compounds **4** and **5** showed the aglycone fragment ions at *m/z* 329 (Y_0_^−^). The fragment ions (Y_1_^−^) formed by the loss of rhamnose were not observed. Compound **4** produced a relatively higher level of the aglycone fragment ion than compound **5,** suggesting that the rutinose was more readily lost than the neohesperidose in the negative ionization mode (Supplementary Figure S3).

**Fig. 3:**
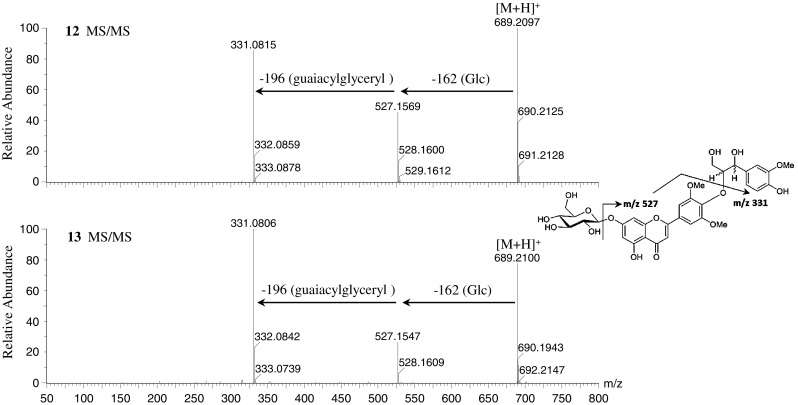
Mass spectra of tricin 4′-*O*-(*erythro*-β-guaiacylglyceryl) ether 7-*O*-β-d-glucopyranoside (**12**) (*m/z* 688) and tricin 4′-*O*-(*threo*-β-guaiacylglyceryl) ether 7-*O*-β-d-glucopyranoside (**13**) (*m/z* 688) at ramped collision energy from 10 to 50 eV in positive ionization mode

The MS/MS spectra of the flavonolignans tricin 4′-*O*-(*erythro*-β-guaiacylglyceryl) ether (**10**) and tricin 4′-*O*-(*threo*-β-guaiacylglyceryl) ether (**11**) in the positive ionization mode showed the same protonated molecular ions at *m/z* 527. Both MS/MS spectra showed major fragment ions of aglycone at *m/z* 331 [(M + H)-196]^+^, corresponding to the loss of a guaiacylglyceryl group. Similarly, MS/MS data of tricin 4′-*O*-(*erythro*-β-guaiacylglyceryl) ether 7-*O*-β-d-glucopyranoside (**12**) and tricin 4′-*O*-(*threo*-β-guaiacylglyceryl) ether 7-*O*-β-d-glucopyranoside (**13**) showed major product ions at *m/z* 527 [(M + H)-162]^+^, corresponding to the loss of glucose and aglycone fragment ions, and at *m/z* 331 [(M + H)-162-196]^+^, indicating the loss of glucose and guaiacylglyceryl groups (Fig. 3 and Supplementary Figure S4). The MS/MS spectra of tricin 4′-*O*-(*erythro*-β-guaiacylglyceryl) ether 7′′-*O*-β-d-glucopyranoside (**14**), tricin 4′-*O*-(*threo*-β-guaiacylglyceryl) ether 7′′-*O*-β-d-glucopyranoside (**15**), and tricin 4′-*O*-(*erythro*-β-guaiacylglyceryl) ether 9′′-*O*-β-d-glucopyranoside (**16**) showed major aglycone fragment ions at *m/z* 331 and a lower amount of product ions at *m/z* 527. In addition, compounds **14**, **15**, and **16** showed fragment ions at *m/z* 509 [(M + H)-162-18]^+^, which were formed by the loss of one water molecule from ions at *m/z* 527 (Supplementary Figure S4). These results suggested that fragment ions at *m/z* 509 were characteristic fragments of flavonolignans **14**, **15**, and **16**, which have a glucose located at the 7′′- or 9′′-position of the guaiacylglyceryl group. In the negative ionization mode, flavonolignans **10**–**16** also showed similar fragment patterns with neutral loss of guaiacylglyceryl and glucose groups (Supplementary Figure S4).

#### Fragmentation of flavonoid C-glycosides and O,C-glycosides

In the MS/MS spectra, the fragmentation patterns of *C*-glycosides differ from those of *O*-glycosides; loss of water molecules and cross-ring cleavages of sugar residues are characteristic fragments of *C*-glycosides, whereas the neutral loss of a sugar moiety can be observed in *O,C-*glycosides (Cuyckens and Claeys 2004).

The MS/MS spectra of the flavonoid *C*-glycosides apigenin 6-*C*-α-l-arabinosyl-8-*C*-β-l-arabinoside (**20**) and chrysoeriol 6-*C*-α-l-arabinosyl-8-*C*-β-l-arabinoside (**21**) in the positive ionization mode showed the loss of one, two, and three water molecules from precursor ions at *m/z* 535 and 565, leading to product ions at *m/z* 517, 499, and 481, respectively, for compound **20** and product ions at *m/z* 547, 529, and 511, respectively, for compound **21**. The cross-ring cleavage of the sugar residue of *C*-glycoside yielded many characteristic product ions, such as *m/z* 445 (^0.2^X^+^) and 469 (^0.4^X^+^-2H_2_O) of compound **20** as well as *m/z* 475 (^0.2^X^+^) and 499 (^0.4^X^+^-2H_2_O) of compound **21** (Vukics and Guttman 2010). In the negative ionization mode, the MS/MS spectra showed fewer but characteristic products ions such as *m/z* 473 (^0.3^X^−^), 443 (^0.2^X^−^), 383 (^0.3^X^−^-90 or ^0.2^X^−^-60), and 353 (^0.2^X^−^-90) of compound **20** as well as *m/z* 503 (^0.3^X^−^), 473 (^0.2^X^−^), 413 (^0.3^X^−^-90 or ^0.2^X^−^-60), and 383 (^0.2^X^−^-90) of compound **21** (Supplementary Figure S5). These results suggested that the loss of *m/z* 90 (^0.2^X) and 60 (^0.3^X) are characteristics of flavonoid *C*-pentosides (Vukics and Guttman 2010).

**Fig. 4:**
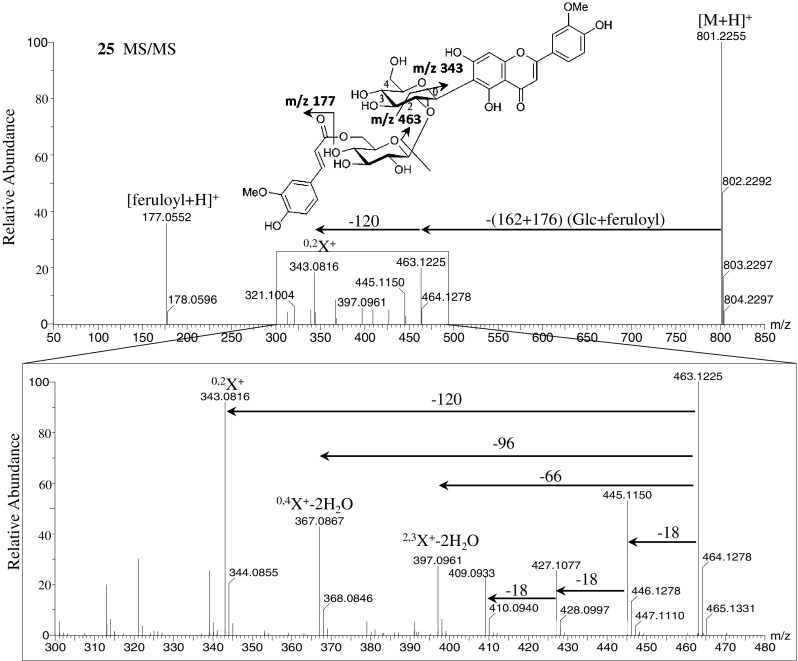
Mass spectra of isoscoparin 2′′-*O*-(6′′′-(*E*)-feruloyl)-glucopyranoside (**25**) (*m/z* 800) at ramped collision energy from 10 to 50 eV in positive ionization mode. The *upper figure* shows the display range at *m/z* 50–850, the *lower figure* shows the expanding range at *m/z* 300–480

The MS/MS spectra of the flavonoid *O,C-*glycosides isoscoparin 2′′-*O*-(6′′′-(*E*)-feruloyl)-glucopyranoside (**25**), isoscoparin 2′′-*O*-(6′′′-(*E*)-*p*-coumaroyl)-glucopyranoside (**26**), isovitexin 2′′-*O*-(6′′′-(*E*)-feruloyl)-glucopyranoside (**27**), and isovitexin 2′′-*O*-(6′′′-(*E*)-*p*-coumaroyl)-glucopyranoside (**28**) in the positive ionization mode showed fragment ions of the *C*-glycoside at *m/z* 463 and 433, which were formed by the neutral loss of glucose and acyl substituents (feruloyl or coumaroyl moiety). Fragment ions of the feruloyl moiety at *m/z* 177 and coumaroyl moiety at *m/z* 147 were also observed (Fig. 4 and Supplementary Figure S6). Compounds **25** and **26** showed fragment ions at *m/z* 445, 427, and 409, which were formed by the loss of water molecules from *C*-glycoside fragment ions at *m/z* 463. Compounds **25** and **26** also gave fragment ions at *m/z* 397 (^2.3^X^+^-2H_2_O), 367 (^0.4^X^+^-2H_2_O), and 343 (^0.2^X^+^), which were formed by the cross-ring cleavage of the sugar residue of the *C*-glycoside fragment at *m/z* 463 (Vukics and Guttman 2010). Compounds **27** and **28** showed similar fragment patterns due to the loss of water molecules and cross-ring cleavages of sugar residues from *C*-glycoside fragments at *m/z* 433 (Supplementary Figure S6). However, the MS/MS spectra of compounds **25**–**28** in the negative ionization mode showed different fragment patterns compared with those for the positive ionization mode (Supplementary Figure S7). Product ions at *m/z* 623 and 593 were formed by the loss of feruloyl or coumaroyl moieties. *C*-glycoside fragment ions at *m/z* 443 and 413 were formed by the neutral loss of glucose and a water molecule from ions at *m/z* 623 and 593, respectively. The MS/MS spectra also showed ferulic acid and coumaric acid ions at *m/z* 193 and 163, respectively. The major fragment ions at *m/z* 323 and 293 (^0.2^X^−^) were formed by cross-ring cleavages of sugar residues of *C*-glycoside fragments at *m/z* 443 and 413, respectively, which are similar to those observed in the positive ion mode. These results suggested that the loss of *m/z* 120 (^0.2^X) is characteristic of flavonoid *C*-hexosides (Waridel et al. 2001; Vukics and Guttman 2010).

## Concluding remarks

Metabolomics aims to identify and quantify all the metabolites in biological samples. The LC–MS/MS approach can generate structural information from precursor and product ions, which can be combined with NMR for unambiguous identification of (un)known phytochemicals. Using this strategy, 36 compounds, including five new flavonoids and eight rare flavonolignan isomers, were isolated and identified from rice. The unique MS/MS fragment patterns of flavonoid *O*-glycosides, *C*-glycosides, and *O,C-*glycosides will facilitate annotation of these plant-specialized metabolites in future studies. Moreover, isolation and structure elucidation of metabolites can enhance the understanding of gene-to-metabolite correlations in phytochemical genomics studies (Nakabayashi et al. 2009; Saito 2013) by integrating metabolomics information with the genomic information. Unequivocal structures of metabolites are also useful for metabolome quantitative trait loci (mQTL) analysis (Matsuda et al. 2012) and genome-wide association studies (GWAS) (Yonemaru et al. 2012). The genomic region and genes potentially responsible for the biosynthesis of specialized metabolites can be presented by mQTL analysis (Matsuda et al. 2012). The obtained compounds and their MS/MS spectra can be used not only for metabolite annotation but also to investigate the relationships between gene expression and metabolite accumulation in rice and other plant metabolic systems.

## Electronic supplementary material

Below is the link to the electronic supplementary material.

Supplementary material 1 (DOCX 663 kb) Procedures for isolation from rice

Supplementary material 2 (DOCX 45 kb) ^1^H- and ^13^C-NMR spectral data of known compounds

Supplementary material 3 (DOCX 207 kb) LC-PDA chromatogram of rice leaf extracts at 340 nm (Figures S1)

Supplementary material 4 (DOCX 2339 kb) MS/MS spectra of isolated compounds (Figures S2-7)

Supplementary material 5 (XLSX 19 kb) MS/MS and on-line UV spectral data of isolated compounds
