# Supplementary material for: Toward better annotation in plant metabolomics: isolation and structure elucidation of 36 specialized metabolites from Oryza sativa (rice) by using MS/MS and NMR analyses
Source: Metabolomics. 2013 Dec 29;10(4):543–55. doi: 10.1007/s11306-013-0619-5 (PMC4097337; doi:10.1007/s11306-013-0619-5)
Supplement: Supplementary file 1 — Supplementary material 1 (DOCX 663 kb) Procedures for isolation from rice [file 11306_2013_619_MOESM1_ESM.docx]

Supplementary data file 1. Procedures for Isolation from *Oryza sativa* (rice)

lyophilized leaf powder of *Oryza sativa* (rice)

（Habataki: HBT）(Total 90 g : dry weight)

Extracted with 90% MeOH overnight

Extracts of 90% MeOH (13.15 g)

Dissolved in water and extracted with n-hexane

water layer

n-hexane layer

Dissolved in 50% MeOH and isolated with Cosmosil 75C18-OPN ODS column (73 x 144 mm),

Eluted with aqueous MeOH containing 0.05% formic acid.

HBT- 　C01

11495.8 mg

C09

485.4 mg

100%

C08

179.9 mg

65%

C07

242.3 mg

50%

C06

327.9 mg

42.5%

C05

148.0 mg

35%

C04

95.2 mg

27.5%

C03

90.3 mg

20%

C02

123.5 mg

12.5%

5%

HBT-C07 (213.5 mg)

HPLC:

Cadenza CD-C18 column (10 x 150 mm, 3 μm),

Solvent: A: Water (0.1% HCOOH); B: MeOH (0.1% HCOOH) 5%B(0–2 min), 5–35% B (2–3 min), 35–40% B (3–21 min), 40–100% B (21–22 min), 100% B (22–27 min); Flow: 3 ml/min; UV: 254 nm; Injection: 100 μL of 70% MeOH sample solution.

HBT-C07- F01

1.8 mg

F10

31.1 mg

F09

12.8 mg

F08

8.8 mg

F07

41.6 mg

F06

9.3 mg

F05

6.3 mg

F04

29.3 mg

F03

9.5 mg

F02

8.1 mg

HBT-C07-F03 (9.5 mg)

HPLC:

Unison UK-C18 column (10 x 150 mm, 3 μm), Oven Tem.40℃

Solvent: A: Water (0.1% HCOOH); B: ACN (0.1% HCOOH) 5%B(0–2 min), 5–19% B (2–3 min), 19–19% B (3–21 min), 19–100% B (21–22 min), 100% B (22–27 min)

Flow: 3.0 ml/min; UV: 254 nm; Injection: 100 μL of 50% MeOH sample solution.

7

1.04 mg

6

0.94 mg

5

0.64 mg

4

0.17 mg

1

0.41 mg

3

0.28 mg

2

0.76 mg

HBT-C07-F03- 0

0.20 mg

HBT-C07-F04 (29.3 mg)

HPLC:

Unison UK-C18 column (10 x 150 mm, 3 μm), Oven Tem.25℃

Solvent: A: Water (0.1% HCOOH); B: ACN (0.1% HCOOH) 5%B(0–2 min), 5–20% B (2–3 min), 20–20% B (3–21 min), 20–100% B (21–22 min), 100% B (22–27 min)

Flow: 3.0 ml/min; UV: 254 nm; Injection: 100 μL of 50% MeOH sample solution.

2

2.20 mg

3

2.04 mg

4

1.25 mg

1

4.28 mg

HBT-C07-F04- 0

0.15 mg

HBT-C07-F05 (6.3 mg)

HPLC:

Unison UK-C18 column (10 x 150 mm, 3 μm),

Solvent: A: Water (0.1% HCOOH); B: ACN (0.1% HCOOH) 5%B(0–2 min), 5–20% B (2–3 min), 20–20% B (3–21 min), 20–100% B (21–22 min), 100% B (22–27 min)

Flow: 3.0 ml/min; UV: 254 nm; Injection: 100 μL of 75% MeOH sample solution.

9

0.43 mg

8

0.11 mg

7

0.29 mg

6

0.19 mg

5

2.07 mg

4

0.15 mg

3

0.09 mg

2

0.14 mg

HBT-C07-F05- 1

0.11 mg

HBT-C07-F06 (9.3 mg)

HPLC:

Unison UK-C18 column (10 x 150 mm, 3 μm), Oven Tem.40℃

Solvent: A: Water (0.1% HCOOH); B: ACN (0.1% HCOOH) 5%B(0–2 min), 5–20% B (2–3 min), 20–20% B (3–21 min), 20–100% B (21–22 min), 100% B (22–27 min)

Flow: 3.0 ml/min; UV: 254 nm; Injection: 100 μL of 75% MeOH sample solution.

2

0.27 mg

7

2.57 mg

5

0.06 mg

6

0.12 mg

4

0.12 mg

3

0.19 mg

8

0.32 mg

10

1.48 mg

9

0.10 mg

HBT-C07-F06- 1

0.35 mg

Compound **2** (9.96 mg) was firstly crystallized from 80% MeOH solution of HBT-C07-F07, and then the residue was isolated by semi-preparative HPLC.

HBT-C07-F07 (31.6 mg)

HPLC:

Unison UK-C18 column (10 x 150 mm, 3 μm), Oven Tem.40℃

Solvent: A: Water (0.1% HCOOH); B: ACN (0.1% HCOOH) 5%B(0–2 min), 5–21% B (2–3 min), 21–21% B (3–21 min), 21–100% B (21–22 min), 100% B (22–28 min)

Flow: 3.0 ml/min; UV: 254 nm; Injection: 100 μL of 80% MeOH sample solution.

10

4.85 mg

9

3.11 mg

7

2.73 mg

5

0.17 mg

6

0.12 mg

4

0.10 mg

3

0.90 mg

8

0.82 mg

2

0.41 mg

HBT-C07-F07- 1

0.55 mg

HBT-C07-F08 (8.8 mg)

HPLC:

Unison UK-C18 column (10 x 150 mm, 3 μm), Oven Tem.40℃

Solvent: A: Water (0.1% HCOOH); B: ACN (0.1% HCOOH) 5%B(0–2 min), 5–21% B (2–3 min), 21–21% B (3–22 min), 21–100% B (22–23 min), 100% B (23–28 min)

Flow: 3.0 ml/min; UV: 254 nm; Injection: 100 μL of 80% MeOH sample solution.

9

2.41 mg

7

0.19 mg

5

0.09 mg

6

1.15 mg

4

0.04 mg

3

1.01 mg

8

0.11 mg

2

0.16 mg

HBT-C07-F08- 1

0.07 mg

HBT-C07-F09 (12.8 mg)

HPLC:

Unison UK-C18 column (10 x 150 mm, 3 μm), Oven Tem.40℃

Solvent: A: Water (0.1% HCOOH); B: ACN (0.1% HCOOH) 5%B(0–2 min), 5–23% B (2–3 min), 23–23% B (3–21 min), 23–100% B (21–22 min), 100% B (22–27 min)

Flow: 3.0 ml/min; UV: 254 nm; Injection: 100 μL of 80% MeOH sample solution.

2

0.33 mg

7

0.72 mg

5

0.87 mg

6

0.37 mg

4

0.65 mg

3

0.04 mg

8

3.93 mg

10

1.12 mg

9

0.28 mg

HBT-C07-F09- 1

0.19 mg

HBT-C07-F08の分取

HBT-C04 (95.2 mg)

HPLC:

Cadenza CD-C18 column (10 x 150 mm, 3 μm), Oven Tem.40℃

Solvent: A: Water (0.1% HCOOH); B: MeOH (0.1% HCOOH) 5% B(0–2 min), 5–18% B (2–3 min), 18–18% B (3–21 min), 18–100% B (21–22 min), 100% B (22–27 min); 　Flow: 3 ml/min; UV: 254 nm; Injection: 100 μL of 30% MeOH sample solution.

HBT-C04- 01

2.44 mg

10

17.51 mg

09

3.87 mg

08

4.99 mg

07

0.97 mg

06

4.84 mg

05

3.59 mg

04

8.93 mg

03

11.36 mg

02

5.42 mg

HBT-C04-4 (8.93 mg)

HPLC:

Unison UK-C18 column (10 x 150 mm, 3 μm), Oven Tem.40℃

Solvent: A: Water (0.1% HCOOH); B: AcCN (0.1% HCOOH) 5% B(0–2 min), 5–9% B (2–3 min), 9–9% B (3–21 min), 9–100% B (21–22 min), 100% B (22–27 min); 　Flow: 3 ml/min; UV: 254 nm; Injection: 100 μL of 30% MeOH sample solution.

08

0.59 mg

07

2.70 mg

06

0.71 mg

05

0.15 mg

04

0.13 mg

03

0.63 mg

02

0.38 mg

HBT-C04-4- 01

0.29 mg

HBT-C04-3 (11.36 mg)

HPLC:

Unison UK-C18 column (10 x 150 mm, 3 μm), Oven Tem.40℃

Solvent: A: Water (0.1% HCOOH); B: AcCN (0.1% HCOOH) 5% B(0–2 min), 5–8% B (2–3 min), 8–8% B (3–21 min), 8–100% B (21–22 min), 100% B (22–27 min); 　Flow: 3 ml/min; UV: 254 nm; Injection: 100 μL of 30% MeOH sample solution.

02

0.40 mg

10

2.31 mg

09

0.41 mg

08

0.37 mg

07

0.15 mg

06

1.13 mg

05

0.67 mg

04

0.36 mg

03

2.29 mg

HBT-C04-3- 01

0.16 mg

HBT-C08 (211.0 mg) (HBT-C08 179.9 mg and HBT-C07-F10 31.1 mg)

HPLC:

Cadenza CD-C18 column (10 x 150 mm, 3 μm), Oven Tem.40℃

Solvent: A: Water (0.1% HCOOH); B: MeOH (0.1% HCOOH) 5% B(0–2 min), 5–45% B (2–3 min), 45–55% B (3–21 min), 55–100% B (21–22 min), 100% B (22–27 min); 　Flow: 3 ml/min; UV: 254 nm; Injection: 100 μL of 80% MeOH sample solution.

10

28.50 mg

HBT-C08- 01

3.81 mg

09

20.19 mg

08

9.73 mg

07

10.12 mg

06

10.85 mg

05

19.25 mg

04

8.73 mg

03

18.38 mg

02

24.75 mg

HBT-C08-2 (24.75 mg)

HPLC:

Unison UK-C18 column (10 x 150 mm, 3 μm), Oven Tem.40℃

Solvent: A: Water (0.1% HCOOH); B: AcCN (0.1% HCOOH) 5% B(0–2 min), 5–25% B (2–3 min), 25–25% B (3–21 min), 25–100% B (21–22 min), 100% B (22–27 min); 　Flow: 3 ml/min; UV: 254 nm; Injection: 100 μL of 80% MeOH sample solution.

10

3.01 mg

09

1.82 mg

08

0.54 mg

07

2.74 mg

06

1.01 mg

05

2.30 mg

04

1.72 mg

03

1.93 mg

02

0.43 mg

HBT-C08-2- 01

0.71 mg

HBT-C08-3 (18.38 mg)

HPLC:

Unison UK-C18 column (10 x 150 mm, 3 μm), Oven Tem.40℃

Solvent: A: Water (0.1% HCOOH); B: AcCN (0.1% HCOOH) 5% B(0–2 min), 5–24% B (2–3 min), 24–24% B (3–26 min), 24–100% B (26–27 min), 100% B (27–32 min); 　Flow: 3 ml/min; UV: 254 nm; Injection: 100 μL of 80% MeOH sample solution.

10

4.20 mg

09

0.83 mg

08

1.04 mg

07

1.51 mg

06

1.02 mg

05

1.43 mg

04

1.10 mg

03

1.05 mg

02

0.87 mg

HBT-C08-3- 01

0.80 mg

HBT-C08-4 (8.73 mg)

HPLC:

Unison UK-C18 column (10 x 150 mm, 3 μm), Oven Tem.40℃

Solvent: A: Water (0.1% HCOOH); B: AcCN (0.1% HCOOH) 5% B(0–2 min), 5–26% B (2–3 min), 26–26% B (3–21 min), 26–100% B (21–22 min), 100% B (22–27 min); 　Flow: 3 ml/min; UV: 254 nm; Injection: 100 μL of 80% MeOH sample solution.

10

1.62 mg

09

0.53 mg

08

1.51 mg

07

0.59 mg

06

0.31 mg

05

0.13 mg

04

0.23 mg

03

0.43 mg

02

0.33 mg

HBT-C08-4- 01

0.34 mg

HBT-C08-5 (19.25 mg)

HPLC:

Unison UK-C18 column (10 x 150 mm, 3 μm), Oven Tem.40℃

Solvent: A: Water (0.1% HCOOH); B: AcCN (0.1% HCOOH) 5% B(0–2 min), 5–27% B (2–3 min), 27–36% B (3–21 min), 36–100% B (21–22 min), 100% B (22–27 min); 　Flow: 3 ml/min; UV: 254 nm; Injection: 100 μL of 80% MeOH sample solution.

10

0.84 mg

09

0.34 mg

08

0.66 mg

07

1.57 mg

06

2.01 mg

05

1.72 mg

04

1.89 mg

03

0.61 mg

02

1.37 mg

HBT-C08-5- 01

3.77 mg

HBT-C08-6 (10.85 mg)

HPLC:

Unison UK-C18 column (10 x 150 mm, 3 μm), Oven Tem.40℃

Solvent: A: Water (0.1% HCOOH); B: AcCN (0.1% HCOOH) 5% B(0–2 min), 5–27% B (2–3 min), 27–27% B (3–21 min), 27–100% B (21–22 min), 100% B (22–27 min); 　Flow: 3 ml/min; UV: 254 nm; Injection: 100 μL of 80% MeOH sample solution.

10

2.83 mg

09

0.45 mg

08

0.37 mg

07

0.71 mg

06

0.58 mg

05

1.23 mg

04

0.56 mg

03

0.75 mg

02

0.41 mg

HBT-C08-6- 01

0.79 mg

HBT-C08-7 (10.12 mg) Oven Tem.40℃

HPLC:

Unison UK-C18 column (10 x 150 mm, 3 μm),

Solvent: A: Water (0.1% HCOOH); B: AcCN (0.1% HCOOH) 5% B(0–2 min), 5–27% B (2–3 min), 27–42% B (3–21 min), 42–100% B (21–22 min), 100% B (22–27 min); 　Flow: 3 ml/min; UV: 254 nm; Injection: 100 μL of 80% MeOH sample solution.

10

0.23 mg

09

0.34 mg

08

0.37 mg

07

0.40 mg

06

1.59 mg

05

0.70 mg

04

0.49 mg

03

0.57 mg

02

0.96 mg

HBT-C08-7- 01

1.52 mg

HBT-C08-8 (9.73 mg)

HPLC:

Unison UK-C18 column (10 x 150 mm, 3 μm), Oven Tem.40℃

Solvent: A: Water (0.1% HCOOH); B: AcCN (0.1% HCOOH) 5% B(0–2 min), 5–34.5% B (2–3 min), 34.5–34.5% B (3–22 min), 34.5–100% B (22–23 min), 100% B (23–28 min); 　Flow: 3 ml/min; UV: 254 nm; Injection: 100 μL of 80% MeOH sample solution.

10

0.74 mg

09

0.59 mg

08

0.51 mg

07

0.97 mg

06

0.64 mg

05

0.38 mg

04

0.65 mg

03

0.29 mg

02

0.86 mg

HBT-C08-8- 01

1.49 mg

HBT-C08-9 (20.19 mg)

HPLC:

Unison UK-C18 column (10 x 150 mm, 3 μm), Oven Tem.40℃

Solvent: A: Water (0.1% HCOOH); B: MeOH (0.1% HCOOH) 5% B(0–2 min), 5–55% B (2–3 min), 55–55% B (3–21 min), 55–100% B (21–22 min), 100% B (22–27 min); 　Flow: 3 ml/min; UV: 254 nm; Injection: 100 μL of 80% MeOH sample solution.

09

0.65 mg

08

4.52 mg

07

2.53 mg

06

3.49 mg

05

1.55 mg

04

1.29 mg

03

0.78 mg

02

0.20 mg

HBT-C08-9- 01

0.93 mg

HBT-C08-10 (28.50 mg)

HPLC:

Unison UK-C18 column (10 x 150 mm, 3 μm), Oven Tem.40℃

Solvent: A: Water (0.1% HCOOH); B: AcCN (0.1% HCOOH) 5% B(0–2 min), 5–40% B (2–3 min), 40–40% B (3–21 min), 40–100% B (21–22 min), 100% B (22–27 min); 　Flow: 3 ml/min; UV: 254 nm; Injection: 100 μL of 80% MeOH sample solution.

10

0.41 mg

09

0.23 mg

08

0.44mg

07

1.31 mg

06

0.27 mg

05

0.32 mg

04

1.63 mg

03

0.22 mg

02

6.40 mg

HBT-C08-10- 01

6.06 mg

HBT-C08分取
