# Supplementary material for: Toward better annotation in plant metabolomics: isolation and structure elucidation of 36 specialized metabolites from Oryza sativa (rice) by using MS/MS and NMR analyses
Source: Metabolomics. 2013 Dec 29;10(4):543–55. doi: 10.1007/s11306-013-0619-5 (PMC4097337; doi:10.1007/s11306-013-0619-5)
Supplement: Supplementary file 2 — Supplementary material 2 (DOCX 45 kb) 1H- and 13C-NMR spectral data of known compounds [file 11306_2013_619_MOESM2_ESM.docx]

Supplementary data file S2. ^1^H- and ^13^C-NMR spectral data of known compounds **1**-**5**, **10**-**23** and **25**-**36**

Tricin (**1**)

^1^H-NMR (600 MHz, DMSO-*d*_6_, 25°C): *δ* 6.98 (1H, s, H-3), 6.20 (1H, d, *J* = 2.4 Hz, H-6), 6.56 (1H, d, *J* = 2.4 Hz, H-8), 7.33 (2H, s, H-2',6'), 3.88 (6H, s, 3',5'-OMe), 12.97 (1H, s, 5-OH). ^13^C-NMR (150 MHz, DMSO-*d*_6_, 25°C): *δ* 164.1 (C-2), 103.6 (C-3), 181.8 (C-4), 103.7 (C-4a), 161.4 (C-5), 98.8 (C-6), 163.6 (C-7), 94.2 (C-8), 157.3 (C-8a), 120.4 (C-1'), 104.4 (C-2',6'), 148.2 (C-3',5'), 139.8 (C-4'), 56.3 (3',5'-OMe).

Tricin 7-*O*-*β*-D-glucopyranoside (**2**)

^1^H-NMR (600 MHz, DMSO-*d*_6_, 25°C): *δ* 7.08 (1H, s, H-3), 6.46 (1H, d, *J* = 2.0 Hz, H-6), 6.94 (1H, d, *J* = 2.0 Hz, H-8), 7.37 (2H, s, H-2',6'), 3.89 (6H, s, 3',5'-OMe), 12.95 (1H, s, 5-OH), 5.06 (1H, d, *J* = 8.2 Hz, H-1''), 3.27 (1H, m, H-2''), 3.30 (1H, m, H-3''), 3.16 (1H, m, H-4''), 3.44 (1H, m, H-5''), 3.73 (1H, dd, *J* = 10.4,5.4 Hz, H-6''), 3.47 (1H, m, H-6''). ^13^C-NMR (150 MHz, DMSO-*d*_6_, 25°C): *δ* 164.1 (C-2), 105.4 (C-3), 182.1 (C-4), 103.8 (C-4a), 161.1 (C-5), 99.5 (C-6), 163.0 (C-7), 95.3 (C-8), 156.9 (C-8a), 120.2 (C-1'), 104.5 (C-2',6'), 148.2 (C-3',5'), 140.0 (C-4'), 56.4 (3',5'-OMe), 100.1 (C-1''), 73.1 (C-2''), 77.4 (C-3''), 69.6 (C-4''), 76.5 (C-5''), 60.6 (C-6'').

Tricin 5-*O*-*β*-D-glucopyranoside (**3**)

^1^H-NMR (600 MHz, DMSO-*d*_6_, 25°C): *δ* 6.84 (1H, s, H-3), 6.80 (2H, s, H-6,8), 7.29 (2H, s, H-2',6'), 3.88 (6H, s, 3',5'-OMe), 4.70 (1H, d, *J* = 7.5 Hz, H-1''), 3.76 (1H, d, *J* = 11.6 Hz, H-6''), 3.56 (1H, dd, *J* = 11.5,5.3 Hz, H-6''). ^13^C-NMR (150 MHz, DMSO-*d*_6_, 25°C): *δ* 161.0 (C-2), 106.3 (C-3), 177.1 (C-4), 107.9 (C-4a), 158.6 (C-5), 104.6 (C-6), 161.0 (C-7), 98.6 (C-8), 158.4 (C-8a), 120.4 (C-1'), 104.0 (C-2',6'), 148.2 (C-3',5'), 139.4 (C-4'), 56.3 (3',5'-OMe), 104.6 (C-1''), 73.6 (C-2''), 75.6 (C-3''), 69.6 (C-4''), 77.5 (C-5''), 60.8 (C-6'').

Tricin 7-*O*-rutinoside (**4**)

^1^H-NMR (600 MHz, DMSO-*d*_6_, 25°C): *δ* 7.06 (1H, s, H-3), 6.48 (1H, d, *J* = 2.2 Hz, H-6), 6.86 (1H, d, *J* = 2.2 Hz, H-8), 7.36 (2H, s, H-2',6'), 3.89 (6H, s, 3',5'-OMe), 5.08 (1H, d, *J* = 7.6 Hz, H-1''), 4.55 (1H, s, H-1'''), 1.07 (3H, d, *J* = 6.2 Hz, H-6'''). ^13^C-NMR (150 MHz, DMSO-*d*_6_, 25°C): *δ* 164.1 (C-2), 103.6 (C-3), 181.9 (C-4), 105.3 (C-4a), 161.2 (C-5), 99.2 (C-6), 162.8 (C-7), 95.2 (C-8), 156.7 (C-8a), 119.9 (C-1'), 104.5 (C-2',6'), 148.1 (C-3',5'), 140.2 (C-4'), 56.3 (3',5'-OMe), 100.4 (C-1''), 73.0 (C-2''), 75.5 (C-3''), 71.9 (C-4''), 76.2 (C-5''), 65.9 (C-6''), 99.8 (C-1'''), 69.5 (C-2'''), 70.2 (C-3'''), 70.6 (C-4'''), 68.2 (C-5'''), 17.7 (C-6''').

Tricin 7-*O*-neohesperidoside (**5**)

^1^H-NMR (600 MHz, DMSO-*d*_6_, 25°C): *δ* 7.06 (1H, s, H-3), 6.38 (1H, d, *J* = 2.0 Hz, H-6), 6.88 (1H, d, *J* = 2.0 Hz, H-8), 7.33 (2H, s, H-2',6'), 3.89 (6H, s, 3',5'-OMe), 5.21 (1H, d, *J* = 7.6 Hz, H-1''), 5.13 (1H, s, H-1'''), 1.20 (3H, d, *J* = 6.2 Hz, H-6'''). ^13^C-NMR (150 MHz, DMSO-*d*_6_, 25°C): *δ* 164.1 (C-2), 103.7 (C-3), 181.9 (C-4), 105.4 (C-4a), 160.9 (C-5), 98.1 (C-6), 162.5 (C-7), 94.8 (C-8), 156.8 (C-8a), 119.8 (C-1'), 104.4 (C-2',6'), 148.2 (C-3',5'), 56.3 (3',5'-OMe), 99.4 (C-1''), 77.1 (C-2''), 76.3 (C-3''), 71.8 (C-4''), 77.2 (C-5''), 60.4 (C-6''), 100.4 (C-1'''), 69.7 (C-2'''), 70.3 (C-3'''), 70.4 (C-4'''), 68.3 (C-5'''), 17.9 (C-6''').

Tricin 4'-*O*-(*erythro*-*β*-guaiacylglyceryl) ether (**10**)

^1^H-NMR (600 MHz, CD_3_OD, 25°C): *δ* 6.69 (1H, s, H-3), 6.20 (1H, s, H-6), 6.47 (1H, s, H-8), 7.20 (2H, s, H-2',6'), 3.91 (6H, s, 3',5'-OMe), 6.99 (1H, d, *J* = 1.6 Hz, H-2''), 6.73 (1H, d, *J* = 8.1 Hz, H-5''), 6.81 (1H, dd, *J* = 8.1, 1.6 Hz, H-6''), 4.92 (1H, d, *J* = 5.3 Hz, H-5''), 4.45 (1H, m, H-8''), 3.94 (1H, dd, *J* = 12.1, 5.4 Hz, H-9''), 3.68 (1H, dd, *J* = 12.1, 3.4 Hz, H-9''), 3.83 (3H, s, 3''-OMe). ^13^C-NMR (150 MHz, CD_3_OD, 25°C): *δ* 165.3 (C-2), 105.9 (C-3), 183.9 (C-4), 105.5 (C-4a), 163.3 (C-5), 100.3 (C-6), 166.2 (C-7), 95.3 (C-8), 159.5 (C-8a), 127.9 (C-1'), 105.2 (C-2',6'), 154.9 (C-3',5'), 140.7 (C-4'), 57.0 (3',5'-OMe), 133.9 (C-1''), 111.6 (C-2''), 148.7 (C-3''), 147.0 (C-4''), 115.7 (C-5''), 120.9 (C-6''), 74.3 (C-7''), 87.6 (C-8''), 62.0 (C-9''), 56.4 (3''-OMe).

Tricin 4'-*O*-(*threo*-*β*-guaiacylglyceryl) ether (**11**)

^1^H-NMR (600 MHz, CD_3_OD, 25°C): *δ* 6.71 (1H, s, H-3), 6.21 (1H, s, H-6), 6.48 (1H, s, H-8), 7.23 (2H, s, H-2',6'), 3.95 (6H, s, 3',5'-OMe), 7.03 (1H, d, *J* = 1.2 Hz, H-2''), 6.75 (1H, d, *J* = 7.8 Hz, H-5''), 6.88 (1H, dd, *J* = 7.8, 1.2 Hz, H-6''), 5.01 (1H, d, *J* = 6.5 Hz, H-7''), 4.30 (1H, m, H-8''), 3.81 (1H, dd, *J* = 12.0, 4.2 Hz, H-9''), 3.42 (1H, dd, *J* = 12.0, 3.0 Hz, H-9''), 3.84 (3H, s, 3''-OMe). ^13^C-NMR (150 MHz, CD_3_OD, 25°C): *δ* 165.2 (C-2), 106.0 (C-3), 183.9 (C-4), 105.5 (C-4a), 163.2 (C-5), 100.3 (C-6), 166.3 (C-7), 95.3 (C-8), 159.5 (C-8a), 128.0 (C-1'), 105.2 (C-2',6'), 154.8 (C-3',5'), 141.4 (C-4'), 57.0 (3',5'-OMe), 133.6 (C-1''), 111.8 (C-2''), 148.8 (C-3''), 147.3 (C-4''), 115.9 (C-5''), 120.9 (C-6''), 74.5 (C-7''), 88.9 (C-8''), 62.1 (C-9''), 56.4 (3''-OMe).

Tricin 4'-*O*-(*erythro*-*β*-guaiacylglycery) ether 7-*O*-*β*-D-glucopyranoside (**12**)

^1^H-NMR (600 MHz, DMSO-*d*_6_, 25°C): *δ* 7.14 (1H, br s, H-3), 6.48 (1H, d, *J* = 1.8 Hz, H-6), 6.936 (1H, d, *J* = 1.8 Hz, H-8), 7.34 (2H, s, H-2',6'), 3.88 (6H, s, 3',5'-OMe), 6.943 (1H, d, *J* = 1.8 Hz, H-2''), 6.70 (1H, d, *J* = 7.8 Hz, H-5''), 6.75 (1H, dd, *J* = 7.8, 1.8 Hz, H-6''), 4.80 (1H, br s, H-7''), 4.37 (1H, m, H-8''), 3.73 (1H, m, H-9''), 3.50 (1H, m, H-9''), 3.75 (3H, s, 3''-OMe), 5.05 (1H, d, *J* = 7.8 Hz, H-1'''), 3.28 (1H, m, H-2'''), 3.31 (1H, m, H-3'''), 3.17 (1H, m, H-4'''), 3.43 (1H, m, H-5'''), 3.73 (1H, m, H-6'''), 3.47 (1H, m, H-6'''). ^13^C-NMR (150 MHz, DMSO-*d*_6_, 25°C): *δ* 163.5 (C-2), 104.9 (C-3), 182.1 (C-4), 105.4 (C-4a), 161.0 (C-5), 99.5 (C-6), 163.1 (C-7), 95.3 (C-8), 156.8 (C-8a), 124.9 (C-1'), 104.3 (C-2',6'), 152.9 (C-3',5'), 139.6 (C-4'), 56.3 (3',5'-OMe), 133.1 (C-1''), 110.9 (C-2''), 146.9 (C-3''), 145.3 (C-4''), 114.6 (C-5''), 119.3 (C-6''), 72.1 (C-7''), 86.4 (C-8''), 60.1 (C-9''), 55.4 (3''-OMe), 100.1 (C-1'''), 73.0 (C-2'''), 77.3 (C-3'''), 69.6 (C-4'''), 76.4 (C-5'''), 60.5 (C-6''').

Tricin 4'-*O*-(*threo*-*β*-guaiacylglycery) ether 7-*O*-*β*-D-glucopyranoside (**13**)

^1^H-NMR (600 MHz, DMSO-*d*_6_, 25°C): *δ* 7.15 (1H, br s, H-3), 6.48 (1H, d, *J* = 1.8 Hz, H-6), 6.94 (1H, d, *J* = 1.8 Hz, H-8), 7.35 (2H, s, H-2',6'), 3.86 (6H, s, 3',5'-OMe), 6.97 (1H, br s, H-2''), 6.69 (1H, d, *J* = 7.8 Hz, H-5''), 6.79 (1H, d, *J* = 7.8 Hz, H-6''), 4.84 (1H, t, *J* = 4.8 Hz, H-7''), 4.28 (1H, m, H-8''), 3.64 (1H, m, H-9''), 3.48 (1H, m, H-9''), 3.73 (3H, s, 3''-OMe), 5.05 (1H, d, *J* = 7.2 Hz, H-1'''), 3.27 (1H, m, H-2'''), 3.31 (1H, m, H-3'''), 3.17 (1H, m, H-4'''), 3.45 (1H, m, H-5'''), 3.72 (1H, m, H-6'''), 3.46 (1H, m, H-6'''). ^13^C-NMR (150 MHz, DMSO-*d*_6_, 25°C): *δ* 163.4 (C-2), 104.9 (C-3), 182.1 (C-4), 105.4 (C-4a), 161.0 (C-5), 99.5 (C-6), 163.1 (C-7), 95.3 (C-8), 156.8 (C-8a), 125.0 (C-1'), 104.3 (C-2',6'), 152.8 (C-3',5'), 140.0 (C-4'), 56.3 (3',5'-OMe), 132.9 (C-1''), 110.9 (C-2''), 146.8 (C-3''), 145.3 (C-4''), 114.6 (C-5''), 119.3 (C-6''), 71.5 (C-7''), 86.9 (C-8''), 60.3 (C-9''), 55.5 (3''-OMe), 100.1 (C-1'''), 73.0 (C-2'''), 77.3 (C-3'''), 69.5 (C-4'''), 76.4 (C-5'''), 60.5 (C-6''').

Tricin 4'-*O*-(*erythro*-*β*-guaiacylglyceryl) ether 7''-*O*-*β*-D-glucopyranoside (**14**)

^1^H-NMR (600 MHz, CD_3_OD, 25°C): *δ* 6.71 (1H, br s, H-3), 6.22 (1H, d, *J* = 1.4 Hz, H-6), 6.48 (1H, br s, H-8), 7.21 (2H, s, H-2',6'), 3.84 (9H, s, 3',5',3''-OMe), 7.20 (1H, d, *J* = 1.4 Hz, H-2''), 6.78 (1H, d, *J* = 8.0 Hz, H-5''), 6.90 (1H, dd, *J* = 8.0,1.4 Hz, H-6''), 5.28 (1H, d, *J* = 3.8 Hz, H-7''), 4.47 (1H, m, H-8''), 3.90 (1H, dd, *J* = 11.6,6.3 Hz,H-9''), 3.49 (1H, dd, *J* = 11.6,4.9 Hz, H-9''), 4.17 (1H, d, *J* = 7.4 Hz, H-1'''), 3.86 (1H, m, H-6'''), 3.67 (1H, dd, *J* = 12.0,6.0 Hz, H-6'''). ^13^C-NMR (150 MHz, CD_3_OD, 25°C): *δ* 165.5 (C-2), 105.9 (C-3), 183.9 (C-4), 105.5 (C-4a), 163.3 (C-5), 100.3 (C-6), 166.4 (C-7), 95.3 (C-8), 159.6 (C-8a), 127.9 (C-1'), 105.1 (C-2',6'), 154.8 (C-3',5'), 141.2 (C-4'), 56.9 (3',5'-OMe), 130.6 (C-1''), 113.1 (C-2''), 148.8 (C-3''), 147.3 (C-4''), 115.6 (C-5''), 122.1 (C-6''), 77.9 (C-7''), 87.0 (C-8''), 62.0 (C-9''), 56.5 (3''-OMe), 101.0 (C-1'''), 75.2 (C-2'''), 78.1 (C-3'''), 72.0 (C-4'''), 77.9 (C-5'''), 62.9 (C-6''').

Tricin 4'-*O*-(*threo*-*β*-guaiacylglyceryl) ether 7''-*O*-*β*-D-glucopyranoside (**15**)

^1^H-NMR (600 MHz, CD_3_OD, 25°C): *δ* 6.74 (1H, br s, H-3), 6.22 (1H, d, *J* = 1.4 Hz, H-6), 6.49 (1H, br s, H-8), 7.27 (2H, s, H-2',6'), 3.96 (6H, s, 3',5'-OMe), 7.09 (1H, d, *J* = 1.4 Hz, H-2''), 6.75 (1H, d, *J* = 8.2 Hz, H-5''), 6.93 (1H, dd, *J* = 8.2,1.6 Hz, H-6''), 5.17 (1H, d, *J* = 5.8 Hz, H-7''), 4.54 (1H, m, H-8''), 3.71 (1H, dd, *J* = 12.3,4.4 Hz,H-9''), 3.35 (1H, m, H-9''), 3.85 (3H, s, 3''-OMe), 4.58 (1H, d, *J* = 7.7 Hz, H-1'''), 3.74 (1H, m, H-6'''), 3.60 (1H, dd, *J* = 11.6,5.3 Hz, H-6'''). ^13^C-NMR (150 MHz, CD_3_OD, 25°C): *δ* 165.3 (C-2), 106.1 (C-3), 183.9 (C-4), 105.5 (C-4a), 163.3 (C-5), 100.4 (C-6), 166.6 (C-7), 95.3 (C-8), 159.6 (C-8a), 128.2 (C-1'), 105.3 (C-2',6'), 154.8 (C-3',5'), 140.6 (C-4'), 57.1 (3',5'-OMe), 131.8 (C-1''), 112.6 (C-2''), 148.6 (C-3''), 147.3 (C-4''), 115.6 (C-5''), 121.4 (C-6''), 81.9 (C-7''), 87.0 (C-8''), 61.7 (C-9''), 56.5 (3''-OMe), 105.0 (C-1'''), 75.7 (C-2'''), 78.2 (C-3'''), 71.5 (C-4'''), 77.9 (C-5'''), 62.6 (C-6''').

Tricin 4'-*O*-(*threo*-*β*-guaiacylglyceryl) ether 9''-*O*-*β*-D-glucopyranoside (**16**)

^1^H-NMR (600 MHz, CD_3_OD, 25°C): *δ* 6.71 (1H, br s, H-3), 6.22 (1H, d, *J* = 1.7 Hz, H-6), 6.49 (1H, br s, H-8), 7.21 (2H, s, H-2',6'), 3.92 (6H, s, 3',5'-OMe), 7.00 (1H, d, *J* = 1.7 Hz, H-2''), 6.72 (1H, d, *J* = 8.3 Hz, H-5''), 6.81 (1H, dd, *J* = 8.3,1.6 Hz, H-6''), 4.92 (1H, d, *J* = 5.3 Hz, H-7''), 4.69 (1H, m, H-8''), 4.11 (1H, dd, *J* = 11.4,3.3 Hz, H-9''), 3.99 (1H, dd, *J* = 11.4,5.6 Hz, H-9''), 3.81 (3H, s, 3''-OMe), 4.31 (1H, d, *J* = 7.7 Hz, H-1'''), 3.84 (1H, m, H-6'''), 3.62 (1H, dd, *J* = 12.0,5.5 Hz, H-6'''). ^13^C-NMR (150 MHz, CD_3_OD, 25°C): *δ* 165.4 (C-2), 105.9 (C-3), 183.9 (C-4), 105.6 (C-4a), 163.3 (C-5), 100.3 (C-6), 166.3 (C-7), 95.3 (C-8), 159.5 (C-8a), 127.8 (C-1'), 105.3 (C-2',6'), 154.7 (C-3',5'), 140.8 (C-4'), 57.1 (3',5'-OMe), 133.5 (C-1''), 111.8 (C-2''), 148.7 (C-3''), 147.1 (C-4''), 115.7 (C-5''), 121.2 (C-6''), 74.3 (C-7''), 86.2 (C-8''), 70.0 (C-9''), 56.4 (3''-OMe), 105.0 (C-1'''), 75.3 (C-2'''), 78.1 (C-3'''), 71.6 (C-4'''), 77.9 (C-5'''), 62.8 (C-6''').

Tricin 4'-*O*-(threo-*β*-4-hydroxyphenylglyceryl) ether (**17**)

^1^H-NMR (600 MHz, CD_3_OD, 25°C): *δ* 6.74 (1H, s, H-3), 6.23 (1H, s, H-6), 6.49 (1H, s, H-8), 7.27 (2H, s, H-2',6'), 3.96 (6H, s, 3',5'-OMe), 7.25 (1H, d, *J* = 8.7 Hz, H-2'',6''), 7.74 (1H, d, *J* = 8.7 Hz, H-3'',5''), 5.01 (1H, d, *J* = 6.5 Hz, H-7''), 4.27 (1H, m, H-8''), 3.80 (1H, m, H-9''), 3.38 (1H, dd, *J* = 12.3,3.5 Hz, H-9'').

Syringetin 3-*O*-glucopyranoside (**18**)

^1^H-NMR (600 MHz, DMSO-*d*_6_, 25°C): *δ* 6.11 (1H, s, H-6), 6.37 (1H, s, H-8), 7.47 (2H, s, H-2',6'), 3.83 (6H, s, 3',5'-OMe), 5.57 (1H, d, *J* = 7.4 Hz, H-1'').

Syringetin 3-*O*-rutinoside (**19**)

^1^H-NMR (600 MHz, DMSO-*d*_6_, 25°C): *δ* 6.14 (1H, s, H-6), 6.41 (1H, s, H-8), 7.46 (2H, s, H-2',6'), 3.83 (6H, s, 3',5'-OMe), 5.46 (1H, d, *J* = 7.3 Hz, H-1''), 4.43 (1H, d, *J* = 3.0 Hz, H-1'''), 0.97 (3H, d, *J* = 6.1 Hz, H-6''').

Apigenin 6-*C*-*α*-L-arabinosyl-8-*C*-*β*-L-arabinoside (**20**)

^1^H-NMR (600 MHz, DMSO-*d*_6_, 45°C): *δ* 6.62 (1H, s, H-3), 7.94 (2H, d, *J* = 9.0 Hz, H-2',6'), 6.89 (2H, d, *J* = 8.4 Hz, H-3',5'), 13.55 (1H, br s, 5-OH), 4.48 (1H, d, *J* = 9.6 Hz, H-1''), 5.42 (1H, br s, H-1''').

Chrysoeriol 6-*C*-*α*-L-arabinosyl-8-*C*-*β*-L-arabinoside (**21**)

^1^H-NMR (600 MHz, DMSO-*d*_6_, 45°C): *δ* 6.70 (1H, s, H-3), 7.54 (1H, br s, H-2'), 6.90 (1H, d, *J* = 8.5 Hz, H-5'), 7.56 (1H, d, *J* = 8.5 Hz, H-6'), 3.90 (3H, s, 3'-OMe), 13.62 (1H, br s, 5-OH), 4.48 (1H, d, *J* = 9.2 Hz, H-1''), 5.44 (1H, br s, H-1''').

Swertisin (**22**)

^1^H-NMR (600 MHz, DMSO-*d*_6_, 45°C): *δ* 6.82 (2H, s, H-3,8), 7.96 (2H, d, *J* = 8.8 Hz, H-2',6'), 6.94 (2H, d, *J* = 8.7 Hz, H-3',5'), 3.89, 3.87 (3H, s, 7-OMe), 4.59 (1H, d, *J* = 10.2 Hz, H-1''). ^13^C-NMR (150 MHz, DMSO-*d*_6_, 45°C): *δ* 163.9, 163.8 (C-2), 103.0 (C-3), 181.8, 181.6 (C-4), 109.8 (C-6), 164.9, 164.6 (C-7), 90.1, 90.0 (C-8), 156.9, 156.7 (C-8a), 120.9 (C-1'), 128.4 (C-2',6'), 116.0 (C-3',5'), 161.3 (C-4'), 56.4, 56.2 (7-OMe), 72.8, 72.6 (C-1''), 70.9, 70.3 (C-2''), 79.1 (C-3''), 69.7 (C-4''), 81.7, 81.5 (C-5''), 61.8, 60.7 (C-6'').

Isoorientin 7,3'-dimethyl ether (**23**)

^1^H-NMR (600 MHz, DMSO-*d*_6_, 45°C): *δ* 6.92 (1H, s, H-3), 6.83 (1H, s, H-8), 7.59 (1H, d, *J* = 1.9 Hz, H-2'), 7.61 (1H, d, *J* = 8.8 Hz, H-5'), 6.94 (1H, d, *J* = 8.2 Hz, H-6'), 3.90 (3H, s, 3'-OMe), 3.88 (3H, s, 7-OMe), 4.59 (1H, d, *J* = 10.8 Hz, H-1'').

Isoscoparin 2''-*O*-(6'''-(*E*)-feruloyl)-glucopyranoside (**25**)

^1^H-NMR (600 MHz, DMSO-*d*_6_, 45°C): *δ* 6.54 (1H, br s, H-3), 6.40 (1H, br s, H-8), 7.38 (1H, br s, H-2'), 6.86 (1H, d, *J* = 8.4 Hz, H-5'), 7.37 (1H, d, *J* = 6.0 Hz, H-6'), 3.81 (3H, s, 3'-OMe), 4.69 (1H, d, *J* = 9.6 Hz, H-1''), 4.28 (1H, br s, H-1'''), 7.11 (1H, br s, H-2''''), 6.74 (1H, br s, H-5''''), 7.03 (1H, br s, H-6''''), 7.11 (1H, br s, H-7''''), 6.15 (1H, d, *J* = 14.0 Hz, H-8''''), 3.80 (3H, s, 3''''-OMe). ^13^C-NMR (150 MHz, DMSO-*d*_6_, 45°C): *δ* 163.4 (C-2), 103.0 (C-3), 181.8 (C-4), 103.0 (C-4a), 159.6 (C-5), 107.9 (C-6), 163.4 (C-7), 93.7 (C-8), 156.3 (C-8a), 121.7 (C-1'), 110.3 (C-2'), 150.6 (C-3'), 147.8 (C-4'), 115.6 (C-5'), 120.6 (C-6'), 55.8 (3'-OMe), 71.1 (C-1''), 81.5 (C-2''), 78.5 (C-3''), 70.2 (C-4''), 81.5 (C-5''), 61.4 (C-6''), 105.4 (C-1'''), 74.4 (C-2'''), 76.4 (C-3'''), 68.9 (C-4'''), 73.3 (C-5'''), 62.2 (C-6'''), 125.4 (C-1''''), 113.9 (C-2''''), 147.8 (C-3''''), 149.2 (C-4''''), 115.3 (C-5''''), 122.8 (C-6''''), 144.4 (C-7''''), 115.6 (C-8''''), 166.2 (C-9''''), 55.6 (3''''-OMe).

Isoscoparin 2''-*O*-(6'''-(*E*)-*p*-coumaroyl)-glucopyranoside (**26**)

^1^H-NMR (600 MHz, DMSO-*d*_6_, 45°C): *δ* 6.59 (1H, s, H-3), 6.39 (1H, s, H-8), 7.39 (1H, br s, H-2'), 6.86 (1H, d, *J* = 7.0 Hz, H-5'), 7.38 (1H, d, *J* = 8.6 Hz, H-6'), 3.82 (3H, s, 3'-OMe), 4.69 (1H, d, *J* = 9.8 Hz, H-1''), 4.28 (1H, br s, H-1'''), 7.30 (2H, br s, H-2'''',6''''), 6.75 (2H, d, *J* = 7.1 Hz, H-3'''',5''''), 7.17 (1H, d, *J* = 16.1 Hz, H-7''''), 6.10 (1H, d, *J* = 15.7 Hz, H-8''''). ^13^C-NMR (150 MHz, DMSO-*d*_6_, 45°C): *δ* 163.3 (C-2), 103.0 (C-3), 181.7 (C-4), 103.0 (C-4a), 159.6 (C-5), 108.0 (C-6), 163.3 (C-7), 93.9 (C-8), 156.3 (C-8a), 121.6 (C-1'), 110.3 (C-2'), 150.6 (C-3'), 147.9 (C-4'), 115.6 (C-5'), 120.2 (C-6'), 55.8 (3'-OMe), 71.2 (C-1''), 81.5 (C-2''), 78.5 (C-3''), 70.2 (C-4''), 81.5 (C-5''), 61.4 (C-6''), 105.3 (C-1'''), 74.4 (C-2'''), 76.4 (C-3'''), 69.0 (C-4'''), 73.4 (C-5'''), 62.3 (C-6'''), 125.0 (C-1''''), 129.9 (C-2'''',6''''), 115.6 (C-3'''',5''''), 159.6 (C-4''''), 144.1 (C-7''''), 113.7 (C-8''''), 166.1 (C-9'''').

Isovitexin 2''-*O*-(6'''-(*E*)-feruloyl)-glucopyranoside (**27**)

^1^H-NMR (600 MHz, DMSO-*d*_6_, 45°C): *δ* 6.48 (1H, s, H-3), 6.39 (1H, s, H-8), 7.72 (2H, d, *J* = 8.4 Hz, H-2',6'), 6.85 (2H, d, *J* = 7.8 Hz, H-3',5'), 4.69 (1H, d, *J* = 9.6 Hz, H-1''), 4.28 (1H, br s, H-1'''), 7.11 (1H, br s, H-2''''), 6.77 (1H, d, *J* = 7.8 Hz, H-5''''), 6.93 (1H, br s, H-6''''), 7.16 (1H, d, *J* = 15.8 Hz, H-7''''), 6.18 (1H, d, *J* = 15.8 Hz, H-8''''), 3.81 (3H, s, 3''''-OMe). ^13^C-NMR (150 MHz, DMSO-*d*_6_, 45°C): *δ* 163.5 (C-2), 102.6 (C-3), 181.7 (C-4), 103.0 (C-4a), 161.0 (C-5), 108.0 (C-6), 163.5 (C-7), 93.8 (C-8), 156.4 (C-8a), 121.3 (C-1'), 128.2 (C-2',6'), 115.7 (C-3',5'), 163.5 (C-4'), 71.2 (C-1''), 81.5 (C-2''), 78.6 (C-3''), 70.2 (C-4''), 81.5 (C-5''), 61.4 (C-6''), 105.4 (C-1'''), 74.4 (C-2'''), 76.4 (C-3'''), 68.9 (C-4'''), 73.4 (C-5'''), 62.3 (C-6'''), 125.5 (C-1''''), 111.0 (C-2''''), 149.2 (C-3''''), 147.8 (C-4''''), 115.4 (C-5''''), 122.9 (C-6''''), 144.4 (C-7''''), 114.0 (C-8''''), 166.1 (C-9''''), 55.7 (3''''-OMe).

Isovitexin 2''-*O*-(6'''-(*E*)-*p*-coumaroyl)-glucopyranoside (**28**)

^1^H-NMR (600 MHz, DMSO-*d*_6_, 45°C): *δ* 6.50 (1H, s, H-3), 6.37 (1H, s, H-8), 7.73 (2H, d, *J* = 9.0 Hz, H-2',6'), 6.85 (2H, d, *J* = 7.8 Hz, H-3',5'), 4.69 (1H, d, *J* = 10.8 Hz, H-1''), 4.27 (1H, br s, H-1'''), 7.33 (2H, br s, H-2'''',6''''), 6.77 (2H, d, *J* = 7.8 Hz, H-3'''',5''''), 7.20 (1H, d, *J* = 15.9 Hz, H-7''''), 6.11 (1H, d, *J* = 15.9 Hz, H-8''''). ^13^C-NMR (150 MHz, DMSO-*d*_6_, 45°C): *δ* 163.4 (C-2), 102.6 (C-3), 181.6 (C-4), 103.0 (C-4a), 159.6 (C-5), 108.0 (C-6), 163.4 (C-7), 93.8 (C-8), 156.4 (C-8a), 121.3 (C-1'), 128.2 (C-2',6'), 115.7 (C-3',5'), 160.9 (C-4'), 71.2 (C-1''), 81.5 (C-2''), 78.5 (C-3''), 70.2 (C-4''), 81.5 (C-5''), 61.4 (C-6''), 105.3 (C-1'''), 74.4 (C-2'''), 76.4 (C-3'''), 69.0 (C-4'''), 73.4 (C-5'''), 62.4 (C-6'''), 125.0 (C-1''''), 129.9 (C-2'''',6''''), 115.6 (C-3'''',5''''), 159.6 (C-4''''), 144.1 (C-7''''), 113.8 (C-8''''), 166.1 (C-9'''').

1,3-*O*-diferuloylglycerol (**29**)

^1^H-NMR (600 MHz, CD_3_OD, 25°C): *δ* 4.28 (2H, d, *J* = 5.1 Hz, H-1,3), 4.16 (1H, t, *J* = 5.3 Hz, H-2), 7.17 (2H, d, *J* = 1.6 Hz, H-2',2''), 6.79 (2H, dd, *J* = 8.2 Hz, H-5',5''), 7.06 (2H, dd, *J* = 8.2, 1.8 Hz, H-6',6''), 7.66 (2H, d, *J* = 15.8 Hz, H-7',7''), 6.39 (2H, d, *J* = 15.9 Hz, H-8',8''), 3.87 (6H, s, 3',3''-OMe). ^13^C-NMR (150 MHz, CD_3_OD, 25°C): *δ* 66.4 (C-1,3), 68.7 (C-2), 127.7 (C-1',1''), 111.8 (C-2',2''), 150.8 (C-3',3''), 149.4 (C-4',4''), 116.5 (C-5',5''), 124.2 (C-6',6''), 147.3 (C-7',7''), 115.2 (C-8',8''), 169.0 (C-9',9''), 56.5 (3',3''-OMe).

1-*O*-Feruloyl-*β*-D-glucose (**30**)

^1^H-NMR (600 MHz, CD_3_OD, 25°C): *δ* 7.20 (1H, d, *J* = 1.9 Hz, H-2), 6.82 (1H, d, *J* = 1.9 Hz, H-5), 7.10 (1H, dd, *J* = 8.2, 1.9 Hz, H-6), 7.72 (1H, d, *J* = 15.9 Hz, H-7), 6.40 (1H, d, *J* = 15.9 Hz, H-8), 5.58 (1H, d, *J* = 7.9 Hz, H-1'), 3.85 (1H, dd, *J* = 12.1, 2.0 Hz, H-6'), 3.69 (1H, dd, *J* = 12.1, 5.0 Hz, H-6'), 3.89 (3H, s, 3-OMe). ^13^C-NMR (150 MHz, CD_3_OD, 25°C): *δ* 127.6 (C-1), 111.9 (C-2), 149.5 (C-3), 151.0 (C-4), 116.6 (C-5), 124.4 (C-6), 148.3 (C-7), 114.8 (C-8), 167.8 (C-9), 95.9 (C-1'), 74.1 (C-2'), 78.1 (C-3'), 71.2 (C-4'), 78.9 (C-5'), 62.4 (C-6'), 56.5 (3-OMe).

1-*O*-Sinapoyl-*β*-D-glucose (**31**)

^1^H-NMR (600 MHz, CD_3_OD, 25°C): *δ* 6.93 (2H, s, H-2,6), 7.72 (1H, d, *J* = 15.8 Hz, H-7), 6.43 (1H, d, *J* = 15.8 Hz, H-8), 5.58 (1H, d, *J* = 7.9 Hz, H-1'), 3.85 (1H, dd, *J* = 12.1, 2.2 Hz, H-6'), 3.69 (1H, dd, *J* = 12.1, 5.0 Hz, H-6'), 3.88 (6H, s, 3,5-OMe). ^13^C-NMR (150 MHz, CD_3_OD, 25°C): *δ* 126.6 (C-1), 107.2 (C-2,6), 149.6 (C-3,5), 140.0 (C-4), 148.5 (C-7), 115.3 (C-8), 167.7 (C-9), 95.9 (C-1'), 74.1 (C-2'), 78.1 (C-3'), 71.2 (C-4'), 78.9 (C-5'), 62.4 (C-6'), 56.9 (3,5-OMe).

3-*O*-*p*-Coumaroylquinic acid (**32**)

^1^H-NMR (600 MHz, CD_3_OD, 25°C): *δ* 1.94 (2H, m), 2.14 (2H, m), 3.69 (1H, m), 4.09 (1H, m), 5.37 (1H, m, H-3), 6.37 (1H, d, *J* = 15.9 Hz, H-8'), 6.80 (2H, d, *J* = 8.5 Hz, H-3',5'), 7.46 (2H, d, *J* = 8.5 Hz, H-2',6'), 7.65 (1H, d, *J* = 15.9 Hz, H-7'). ^13^C-NMR (150 MHz, CD_3_OD, 25°C): *δ* 37.0, 69.0, 72.9, 74.3, 115.9, 116.8, 127.4, 131.1, 146.4, 161.2, 168.9.

3-*O*-Feruloylquinic acid (**33**)

^1^H-NMR (600 MHz, CD_3_OD, 25°C): *δ* 1.96 (2H, m), 2.16 (2H, m), 3.66 (1H, m), 4.14 (1H, m), 5.37 (1H, m, H-3), 6.81 (1H, d, *J* = 8.1 Hz, H-5'), 7.07 (1H, dd, *J* = 8.2, 1.7 Hz, H-6'), 7.19 (1H, d, *J* = 1.7 Hz, H-2'), 6.40 (1H, d, *J* = 15.9 Hz, H-8'). 7.65 (1H, d, *J* = 15.8 Hz, H-7'). ^13^C-NMR (150 MHz, CD_3_OD, 25°C): *δ* 36.9, 41.5, 56.5, 68.6, 73.1, 74.7, 111.7, 116.3, 116.5, 124.1, 128.0, 146.7, 149.4, 150.5, 168.9.

Salicylic acid 2-*O*-*β*-D-glucopyranoside (**34**)

^1^H-NMR (600 MHz, CD_3_OD, 25°C): *δ* 7.40 (1H, d, *J* = 8.3 Hz, H-3), 7.51 (1H, t, *J* = 7.8 Hz, H-4), 7.13 (1H, t, *J* = 7.4 Hz, H-5), 7.80 (1H, d, *J* = 7.4 Hz, H-6), 4.88 (1H, d, *J* = 7.9 Hz, H-1'), 3.53 (1H, t, *J* = 8.5 Hz, H-2'), 3.48 (1H, m, H-3'), 3.40 (1H, t, *J* = 8.9 Hz, H-4'), 3.46 (1H, m, H-5'), 3.92 (1H, d, *J* = 12.0 Hz, H-6'), 3.72 (1H, dd, *J* = 12.0, 5.6 Hz, H-6'). ^13^C-NMR (150 MHz, CD_3_OD, 25°C): *δ* 123.7 (C-1), 158.8 (C-2), 119.2 (C-3), 134.8 (C-4), 123.9 (C-5), 132.4 (C-6), 104.6 (C-1'), 75.0 (C-2'), 77.5 (C-3'), 71.3 (C-4'), 78.6 (C-5'), 62.6 (C-6'), 170.3 (COOH).

Kynurenic acid (**35**)

^1^H-NMR (600 MHz, DMSO-*d*_6_, 25°C): *δ* 6.43 (1H, s, H-3), 8.02 (1H, d, *J* = 8.0 Hz, H-5), 7.22 (1H, t, *J* = 7.6 Hz, H-6), 7.55 (1H, t, *J* = 7.6 Hz, H-7), 7.93 (1H, d, *J* = 8.3 Hz, H-8). ^13^C-NMR (150 MHz, DMSO-*d*_6_, 25°C): *δ* 139.4 (C-2), 107.3 (C-3), 178.2 (C-4), 139.4 (C-4a), 122.2 (C-5), 119.1 (C-6), 130.9 (C-7), 124.5 (C-8), 125.3 (C-8a), 162.3 (COOH).

Lycoperodine-1 (**36**)

^1^H-NMR (600 MHz, DMSO-*d*_6_, 25°C): *δ* 7.41 (1H, d, *J* = 7.6 Hz, H-4), 6.97 (1H, t, *J* = 7.8 Hz, H-5), 7.04 (1H, t, *J* = 7.6 Hz, H-6), 7.30 (1H, d, *J* = 8.1 Hz, H-7), 10.82 (1H, s, NH).
