# Supplementary material for: Toward better annotation in plant metabolomics: isolation and structure elucidation of 36 specialized metabolites from Oryza sativa (rice) by using MS/MS and NMR analyses
Source: Metabolomics. 2013 Dec 29;10(4):543–55. doi: 10.1007/s11306-013-0619-5 (PMC4097337; doi:10.1007/s11306-013-0619-5)
Supplement: Supplementary file 3 — Supplementary material 3 (DOCX 207 kb) LC-PDA chromatogram of rice leaf extracts at 340 nm (Figures S1) [file 11306_2013_619_MOESM3_ESM.docx]

**32**

**34**

**35**

**33**

**36**

*

**20**

**24**

*

**30**

**31**

**21**

**22**

**6**

**3, 23**

**25-28**

**2**

**12**

**19**

**4**

**5**

**18**

**7**

**13**

**8**

**16**

**14**

**9**

**15**

**1**

**10**

**17**

**11**

**29**

AU

(min)

Figure S1. LC-PDA chromatogram of rice leaves extract at 340 nm. The peak numbers correspond to the isolated compounds (**1**-**36**).

* Isolated compound is not main peak, overlap with other compounds.
