# Supplementary material for: Toward better annotation in plant metabolomics: isolation and structure elucidation of 36 specialized metabolites from Oryza sativa (rice) by using MS/MS and NMR analyses
Source: Metabolomics. 2013 Dec 29;10(4):543–55. doi: 10.1007/s11306-013-0619-5 (PMC4097337; doi:10.1007/s11306-013-0619-5)
Supplement: Supplementary file 4 — Supplementary material 4 (DOCX 2339 kb) MS/MS spectra of isolated compounds (Figures S2-7) [file 11306_2013_619_MOESM4_ESM.docx]

Supplementary Figures S2-7. MS/MS spectra data of isolated compounds from rice


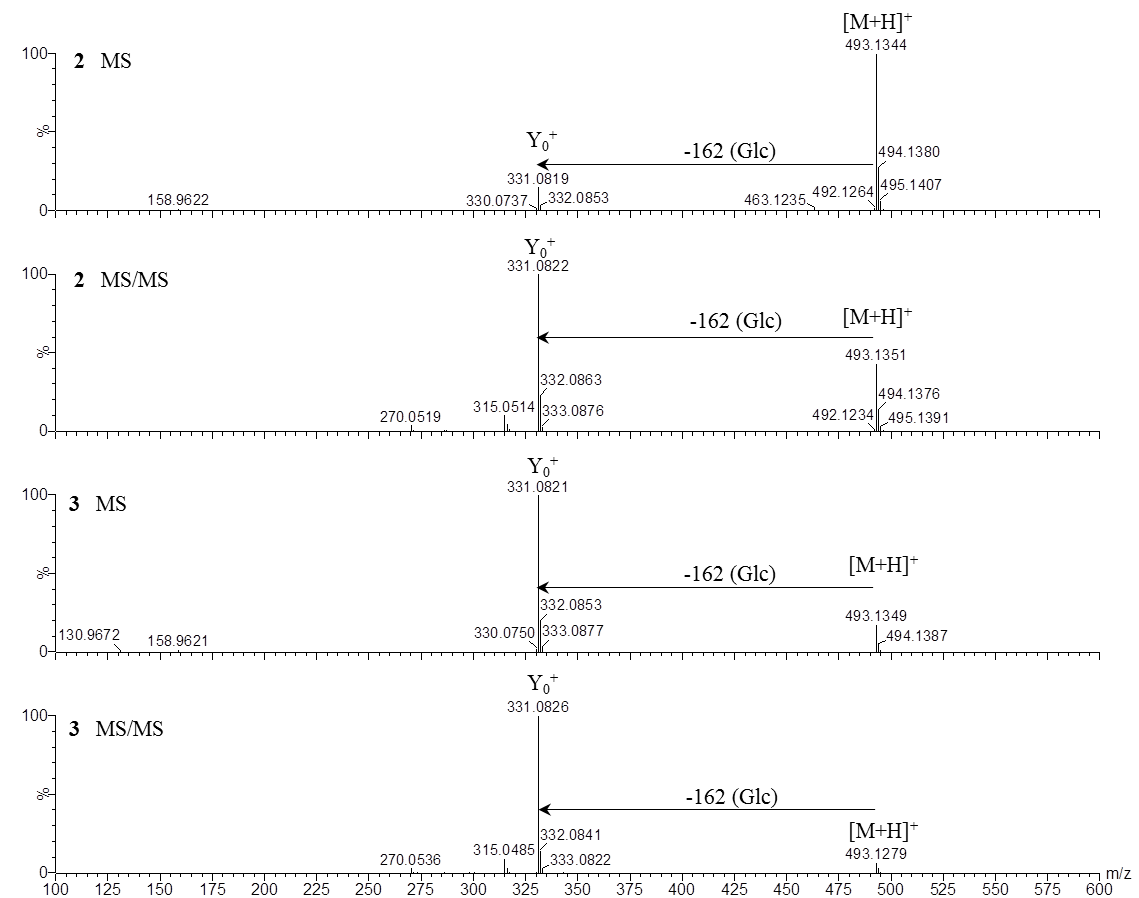


Figure S2. Mass spectra of tricin 7-*O*-*β*-D-glucopyranoside (**2**) (*m/z* 492) and tricin 5-*O*-*β*-D-glucopyranoside (**3**) (*m/z* 492) at ramped collision energy from 10 to 50 eV in positive ionization mode.


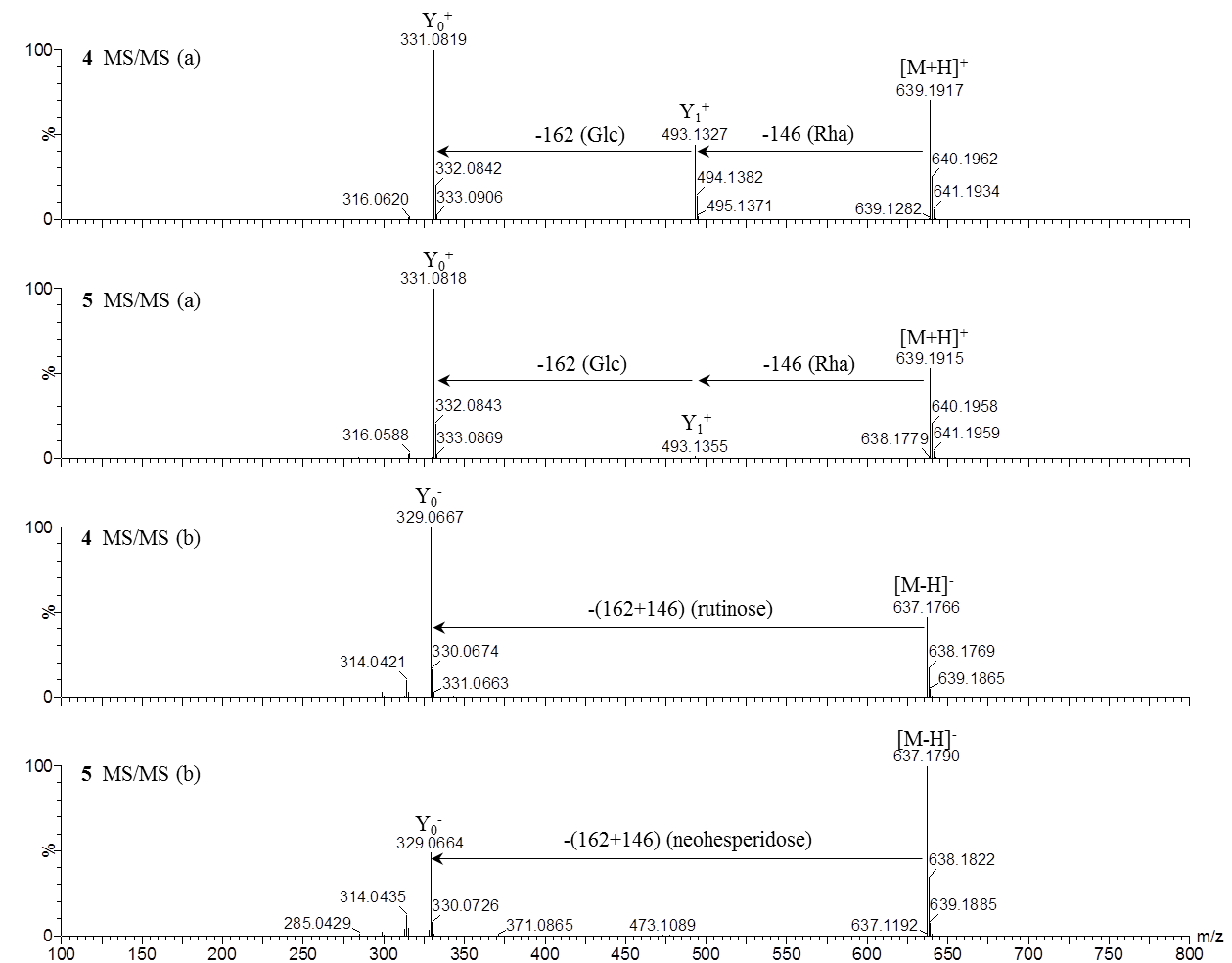


Figure S3. MS/MS spectra of tricin 7-*O*-rutinoside (**4**) (*m/z* 638) and tricin 7-*O*-neohesperidoside (**5**) (*m/z* 638) at ramped collision energy from 10 to 50 eV. (a) positive ionization mode; (b) negative ionization mode.


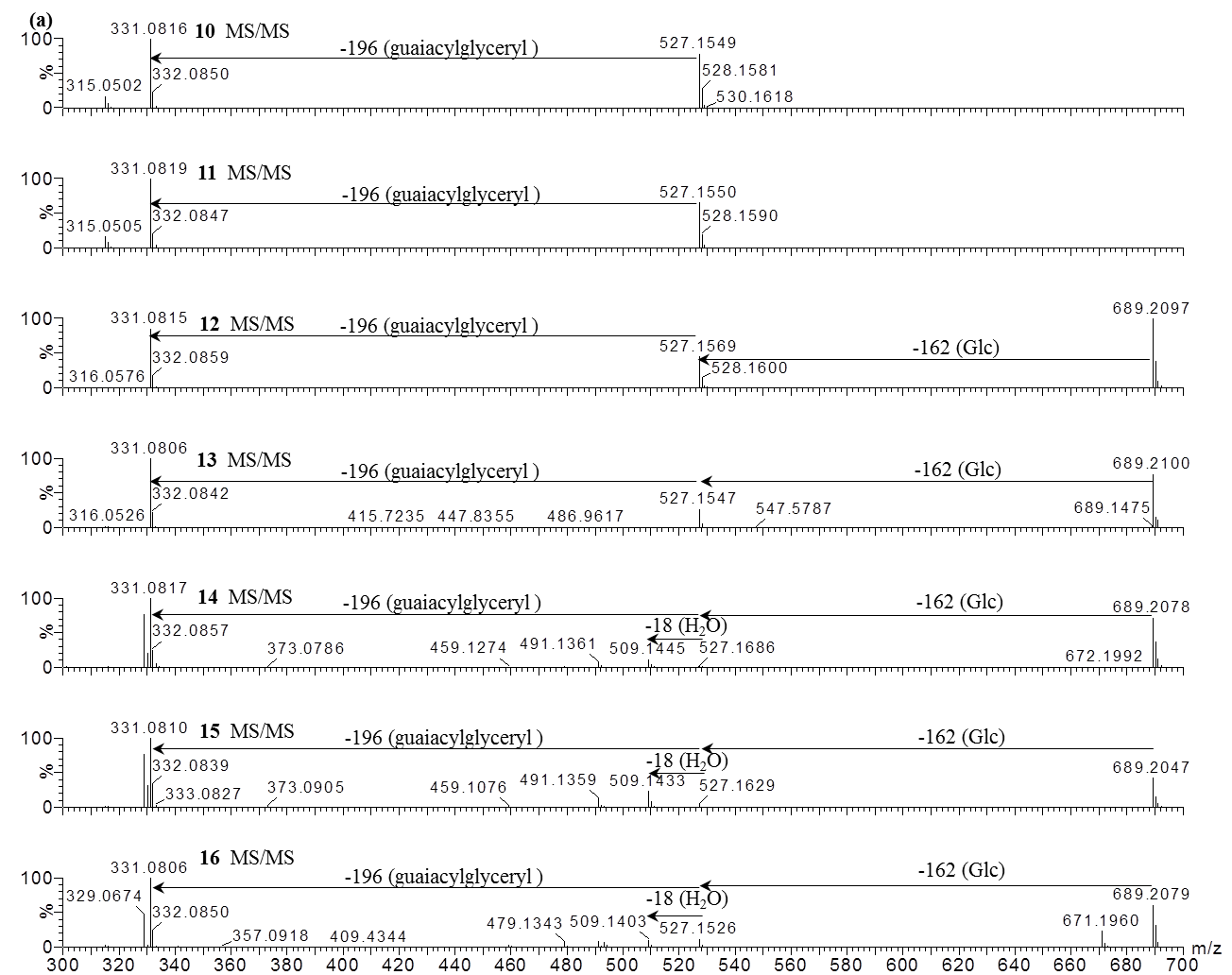


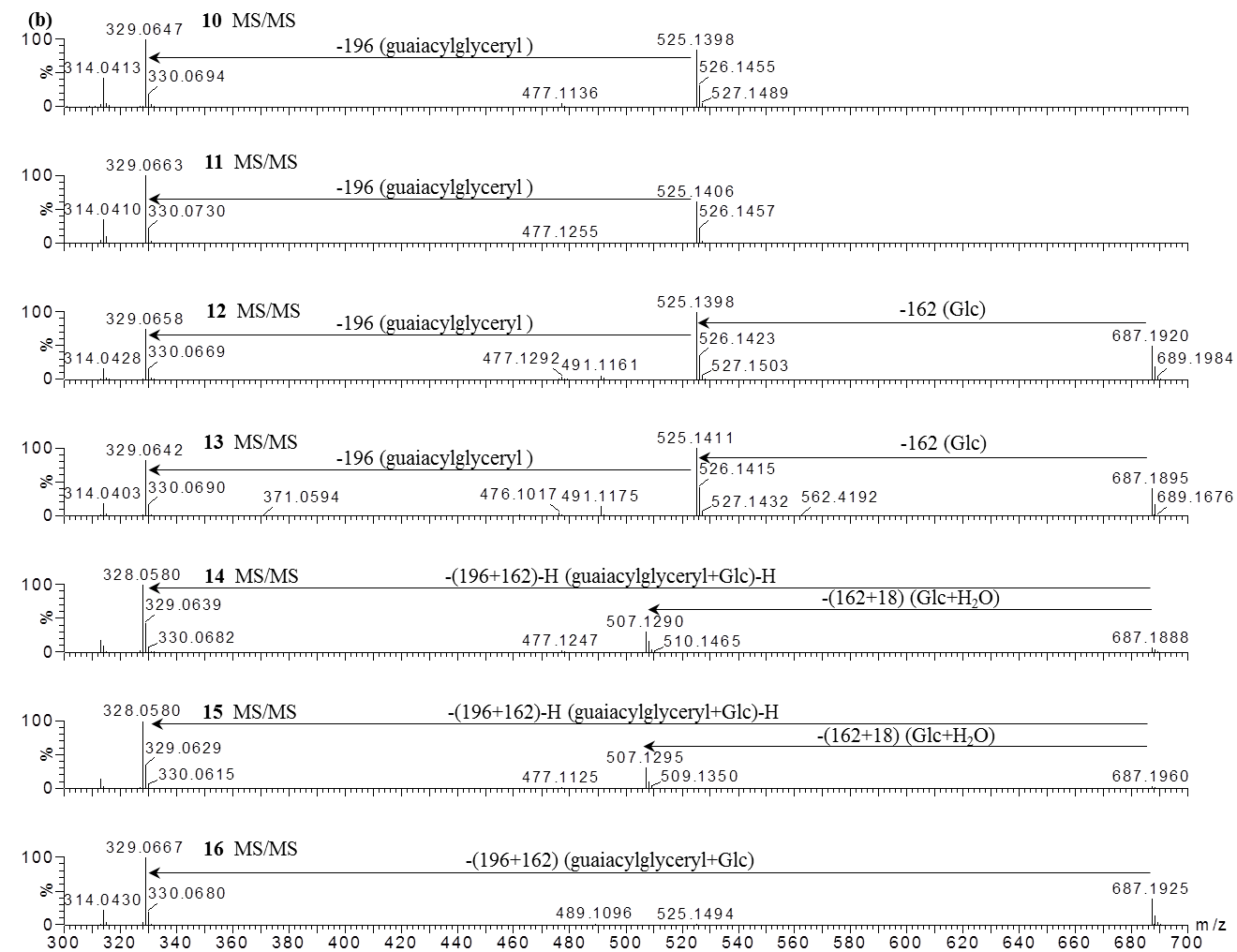


Figure S4. MS/MS spectra of tricin 4'-*O*-(*erythro*-*β*-guaiacylglyceryl) ether (**10**) (*m/z* 526), tricin 4'-*O*-(*threo*-*β*-guaiacylglyceryl) ether (**11**) (*m/z* 526), tricin 4'-*O*-(*erythro*-*β*-guaiacylglyceryl) ether 7-*O*-*β*-D-glucopyranoside (**12**) (*m/z* 688), tricin 4'-*O*-(*threo*-*β*-guaiacylglyceryl) ether 7-*O*-*β*-D-glucopyranoside (**13**) (*m/z* 688), tricin 4'-*O*-(*erythro*-*β*-guaiacylglyceryl) ether 7''-*O*-*β*-D-glucopyranoside (**14**) (*m/z* 688), tricin 4'-*O*-(*threo*-*β*-guaiacylglyceryl) ether 7''-*O*-*β*-D-glucopyranoside (**15**) (*m/z* 688), and tricin 4'-*O*-(*erythro*-*β*-guaiacylglyceryl) ether 9''-*O*-*β*-D-glucopyranoside (**16**) (*m/z* 688) at ramped collision energy from 10 to 50 eV. (a) positive ionization mode; (b) negative ionization mode.


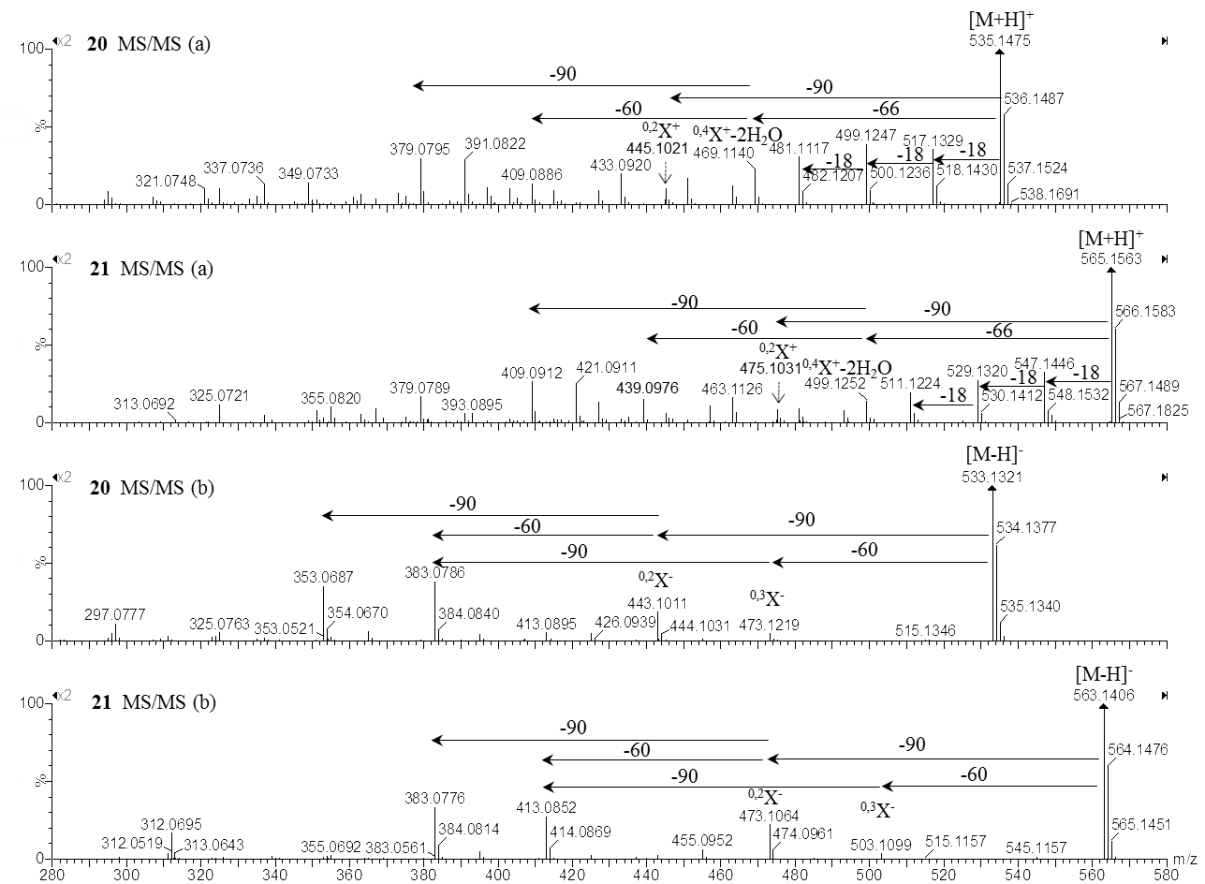


Figure S5. MS/MS spectra (2-fold magnidication) of apigenin 6-*C*-*α*-L-arabinosyl-8-*C*-*β*-L-arabinoside (**20**) (*m/z* 534) and chrysoeriol 6-*C*-*α*-L-arabinosyl-8-*C*-*β*-L-arabinoside (**21**) (*m/z* 564) at ramped collision energy from 10 to 50 eV. (a) positive ionization mode; (b) negative ionization mode.


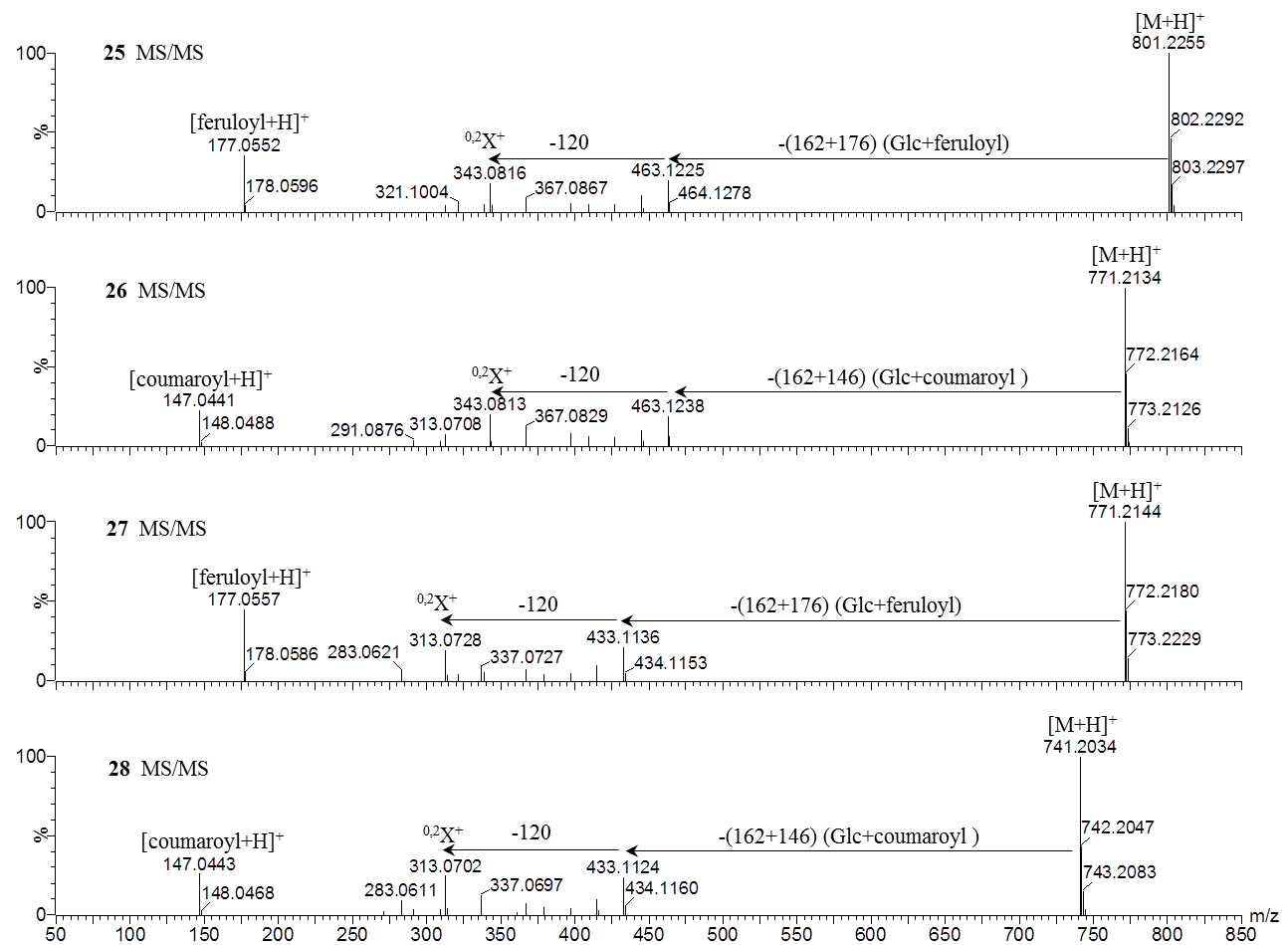


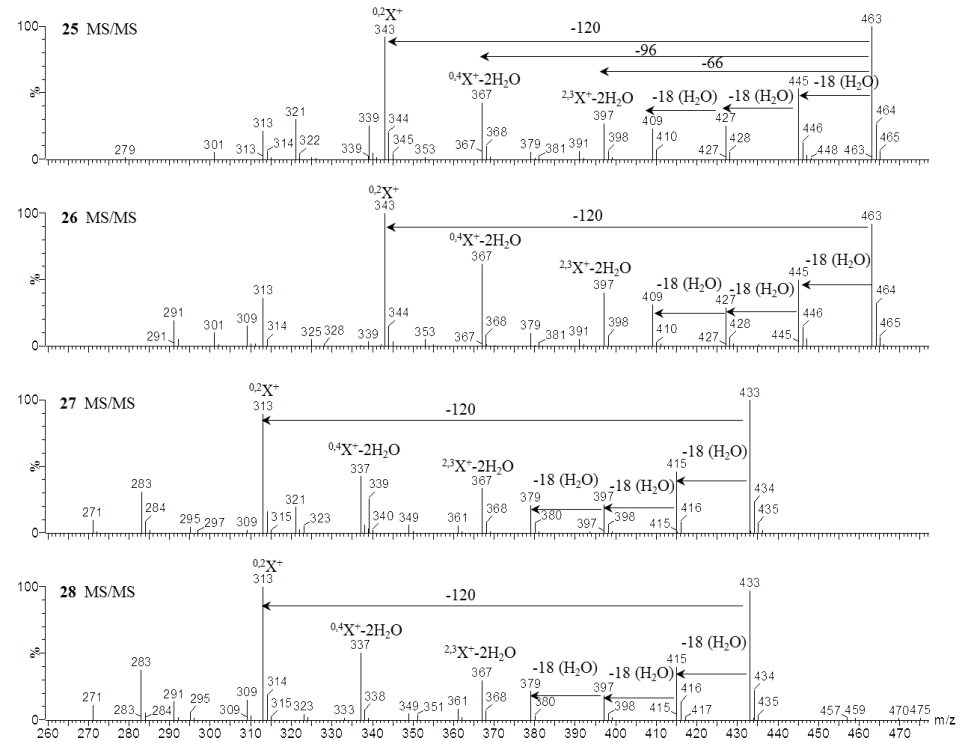


Figure S6. MS/MS spectra of isoscoparin 2''-*O*-(6'''-(*E*)-feruloyl)-glucopyranoside (**25**) (*m/z* 800), isoscoparin 2''-*O*-(6'''-(*E*)-*p*-coumaroyl)-glucopyranoside (**26**) (*m/z* 770), isovitexin 2''-*O*-(6'''-(*E*)- feruloyl)-glucopyranoside (**27**) (*m/z* 770), and isovitexin 2''-*O*-(6'''-(*E*)-*p*-coumaroyl)-glucopyranoside (**28**) (*m/z* 740) at ramped collision energy from 10 to 50 eV in positive ionization mode.


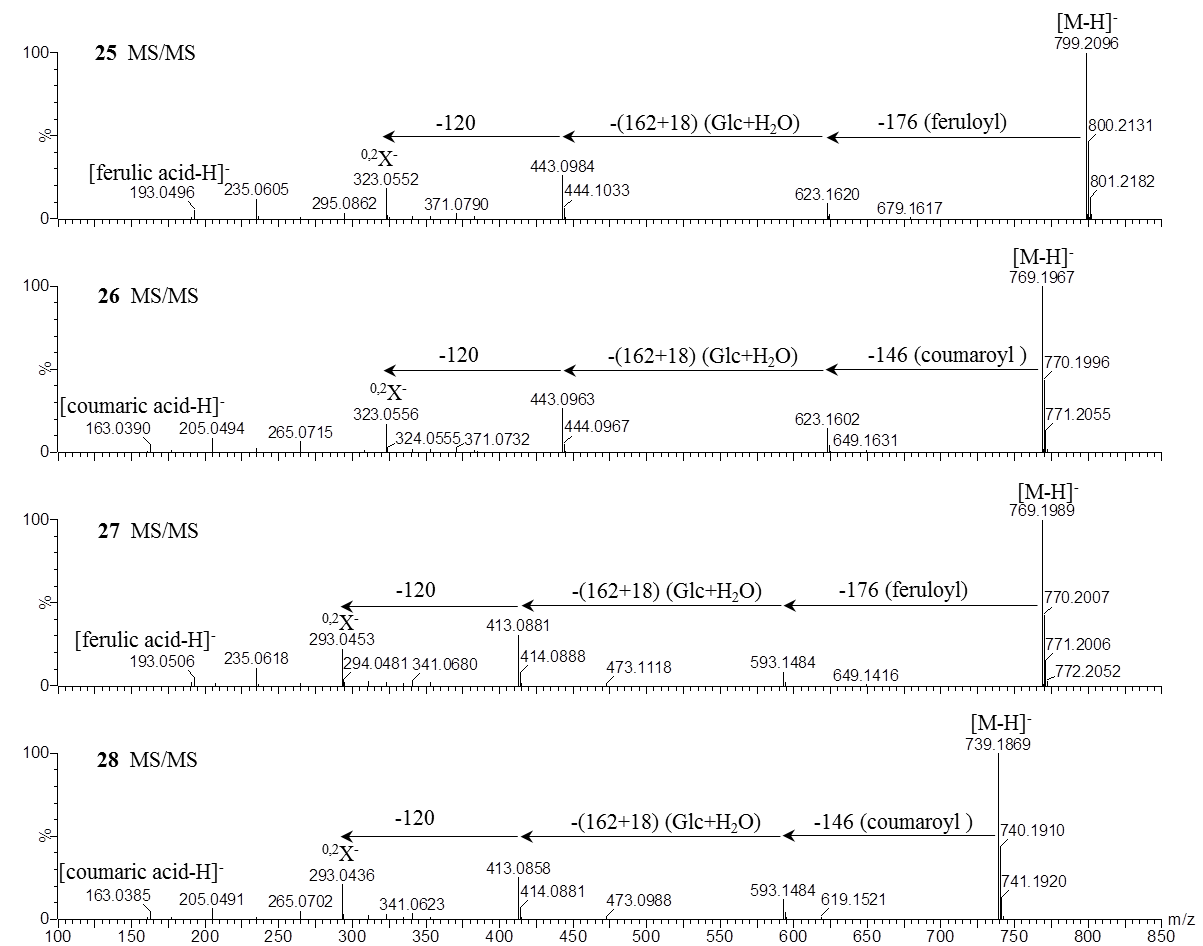


Figure S7. MS/MS spectra of isoscoparin 2''-*O*-(6'''-(*E*)-feruloyl)-glucopyranoside (**25**) (*m/z* 800), isoscoparin 2''-*O*-(6'''-(*E*)-*p*-coumaroyl)-glucopyranoside (**26**) (*m/z* 770), isovitexin 2''-*O*-(6'''-(*E*)- feruloyl)-glucopyranoside (**27**) (*m/z* 770), and isovitexin 2''-*O*-(6'''-(*E*)-*p*-coumaroyl)-glucopyranoside (**28**) (*m/z* 740) at ramped collision energy from 10 to 50 eV in negative ionization mode.
